# Characterisation of the biosynthetic pathway to agnestins A and B reveals the reductive route to chrysophanol in fungi†
†Electronic supplementary information (ESI) available: All experimental and characterisation details. CCDC 1839028 and 1839029. For ESI and crystallographic data in CIF or other electronic format see DOI: 10.1039/c8sc03778g


**DOI:** 10.1039/c8sc03778g

**Published:** 2018-11-26

**Authors:** Agnieszka J. Szwalbe, Katherine Williams, Zhongshu Song, Kate de Mattos-Shipley, Jason L. Vincent, Andrew M. Bailey, Christine L. Willis, Russell J. Cox, Thomas J. Simpson

**Affiliations:** a School of Chemistry , University of Bristol , Cantock's Close , Bristol , BS8 1TS , UK . Email: tom.simpson@bristol.ac.uk; b Syngenta , Jealott's Hill International Research Centre , Bracknell , RG42 6EY , UK; c School of Biological Sciences , 24 Tyndall Avenue , Bristol , BS8 1TQ , UK; d Institute for Organic Chemistry , Leibniz University of Hannover , 30167 , Germany; e BMWZ , Leibniz University of Hannover , 30167 , Germany

## Abstract

Two new dihydroxy-xanthone metabolites, agnestins A and B, were isolated from *Paecilomyces variotii* along with a number of related benzophenones and xanthones including monodictyphenone. The structures were elucidated by NMR analyses and X-ray crystallography. The agnestin (*agn*) biosynthetic gene cluster was identified and targeted gene disruptions of the PKS, Baeyer–Villiger monooxygenase, and other oxido-reductase genes revealed new details of fungal xanthone biosynthesis. In particular, identification of a reductase responsible for *in vivo* anthraquinone to anthrol conversion confirms a previously postulated essential step in aromatic deoxygenation of anthraquinones, *e.g.* emodin to chrysophanol.

## Abstract 2

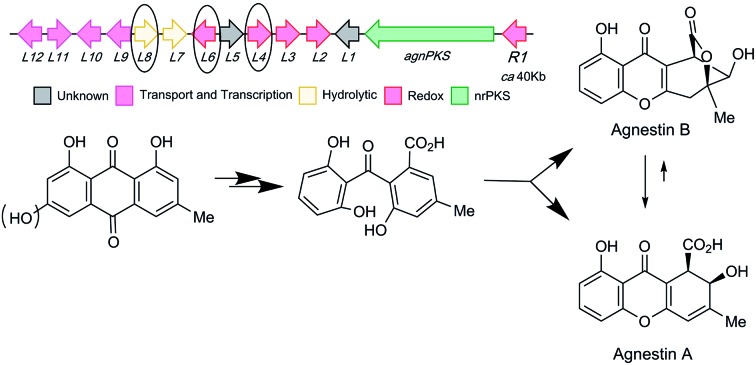
Identification of a reductase (AgnL4) confirms that *in vivo* anthraquinone to anthrol conversion is an essential first step in aromatic deoxygenation of anthraquinones catalysed by AgnL6 (reductase) and AgnL8 (dehydratase).

## 

Xanthones and related benzophenones are produced by a variety of filamentous fungi.^1^ Examples (Scheme 1) include desmethyl-sterigmatocystin **1**,^2^ a key intermediate to the aflatoxin group of mycotoxins produced by *Aspergillus flavus*, ravenelin **2** 3 from *Drechslera ravenelii* and prenylated xanthones, *e.g.* shamixanthone **3** from *Aspergillus variecolor*.^4^ More complex derivatives include the dimeric xanthone ergochromes (secalonic acids) *e.g.***4** from *inter alia Claviceps purpurea*^5^ and *Penicillium oxalicum*.^6^

**Scheme 1:**
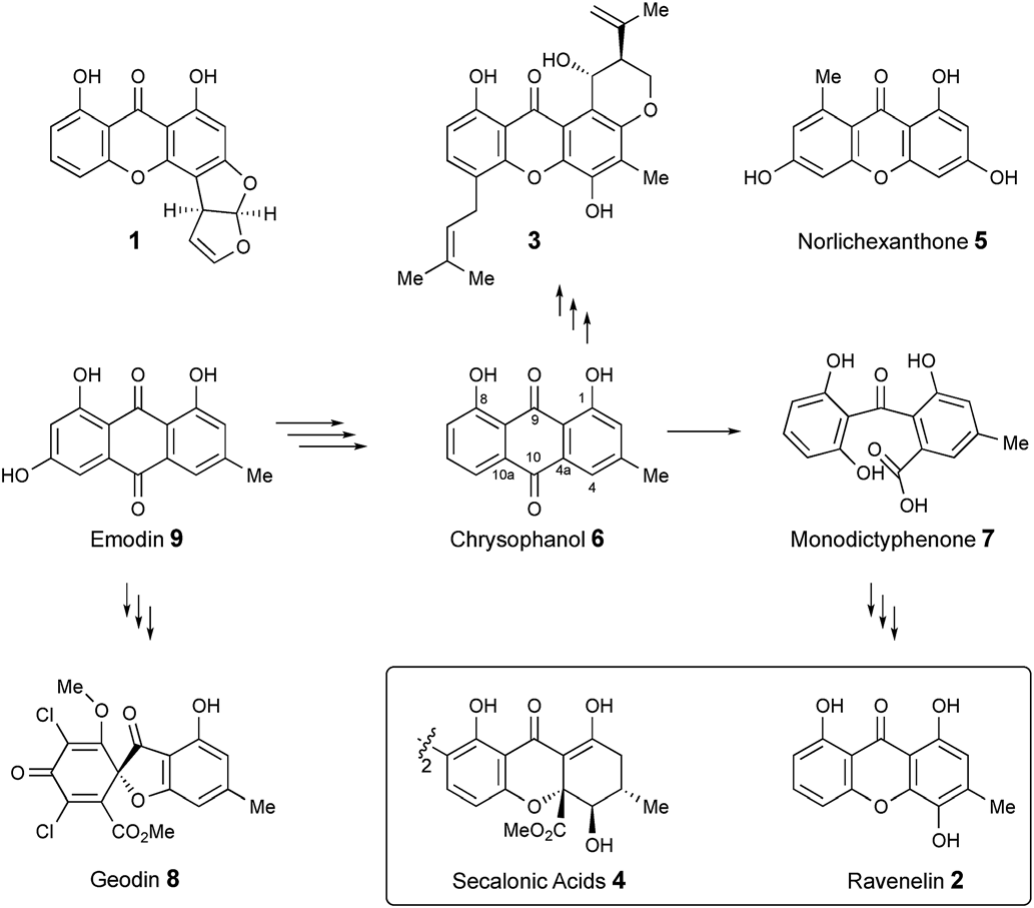
Pathways emanating from emodin **9** and related compounds.

While some fungal xanthones are produced *via* simple folding of a polyketide chain, *e.g.* norlichexanthone **5** from *Lecanora straminea*,^7^ most are produced *via* Baeyer–Villiger-type oxidation of anthraquinones such as chrysophanol **6** to give benzophenones such as monodictyphenone **7** which are subsequently cyclized to xanthones.^8^ Recent interest in these pathways has been stimulated by the results of genome sequencing and bioinformatic analysis^9^ which has allowed identification of the gene clusters in, *e.g. Aspergillus nidulans* for biosynthesis of shamixanthone **3**.^10^,^11^ Other related compounds include geodin **8** 12 which is derived from emodin **9**, the precursor of chrysophanol **6**,^13^ while cladofulvin from the tomato pathogen *Cladosporium fulvum* is a dimeric naphthoquinone also derived from **9**.^14^ Despite these advances key questions remain about the precise genes, proteins and chemical steps involved.

In the course of studies on the biosynthesis of maleidrides,^15^ we examined extracts of the cornexistin producing fungus *Paecilomyces variotii*.^16^ These extracts contained mainly the benzophenone monodictyphenone **7** and other unknown aromatic metabolites. Here we determine the structures of the unknowns, the associated biosynthetic gene cluster and reveal key new redox steps on the fungal pathway to chrysophanol **6**.

## Isolation of metabolites

**Fig. 1:**
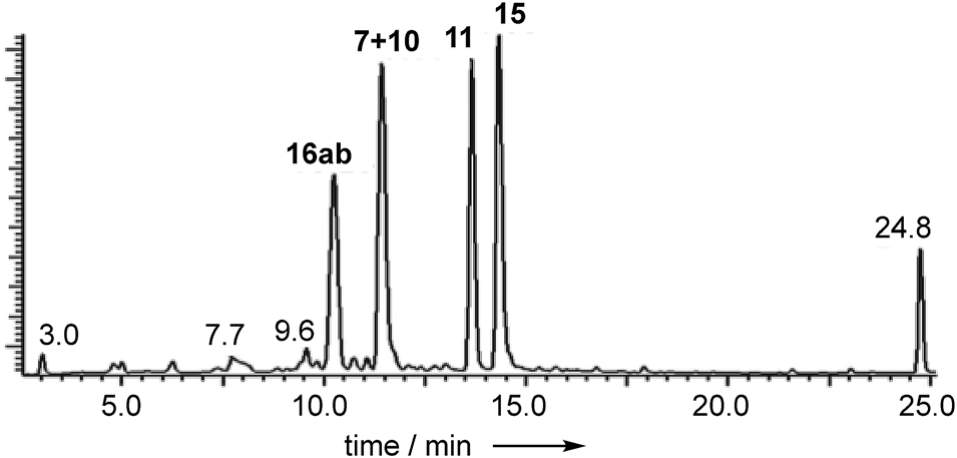
Typical DAD chromatogram of wild type *P. variotii* K5103 culture extract. Bold, compound numbers; other numbers retention times.

Extracts of *P. variotii* K5103 contained several major aromatic metabolites (Fig. 1) which were isolated by semi-preparative HPLC. The material eluting at 11.4 min (typical yield of 20 mg L^–1^ after 15 days fermentation) had ^1^H and ^13^C NMR (see ESI†) and UV spectra which were identical to those for monodictyphenone **7**.^17^ Closer inspection of the ^1^H NMR spectrum revealed the presence (*ca.* 25%) of a co-eluting isomer whose NMR spectra (see ESI†) matched those of the previously reported cephalone F **10** from *Graphiopsis chlorocephala*.^18^ Monodictyphenone **7** is believed to be formed by a Baeyer–Villiger monooxygenase (BVMO) cleavage of the C-10/C-10a bond of chrysophanol **6** and the formation of cephalone F **10** can be rationalized by alternative cleavage at the C-4a/C-10 bond. The reported ^1^H NMR spectrum of cephalone F **10** contained signals due to a minor component (*ca.* 5%) clearly corresponding to those of monodictyphenone **7**, suggesting that the putative BVMO in *G. chlorocephala* has a complementary regioselectivity to that in *P. variotii*.

The peak eluting at 13.6 minutes (typical yields of 15 mg L^–1^) had the same molecular formula as **7**, C_15_H_12_O_6_ (calc. 311.0532, measured 311.0536 for [M + Na]^+^), but whereas the main low resolution mass fragment ion in monodictyphenone **7** occurred at M-44 (–CO_2_), indicating that a carboxylic acid was present, the main fragment ion now occurred at M-62 (–CO_2_, –H_2_O). The NMR spectra also had similarities to monodictyphenone **7**, *e.g.* a methyl group (*δ*_H_ 2.05), a highly conjugated ketone (*δ*_C_ 181.7) and three adjacent aromatic hydrogens (*δ*_H_ 6.91, 7.55 and 6.75) confirmed by their characteristic *ortho* couplings and COSY correlations.

**Fig. 2:**
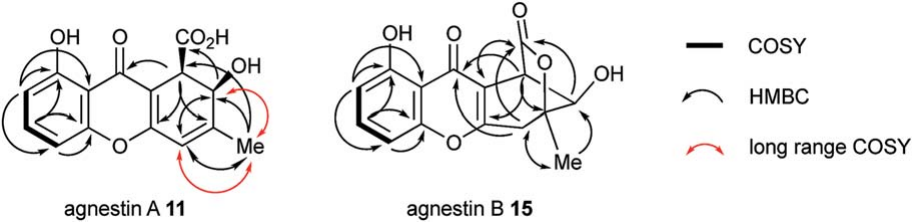
2D correlations (H to C and H to H) observed for agnestin A and agnestin B.

**Fig. 3:**
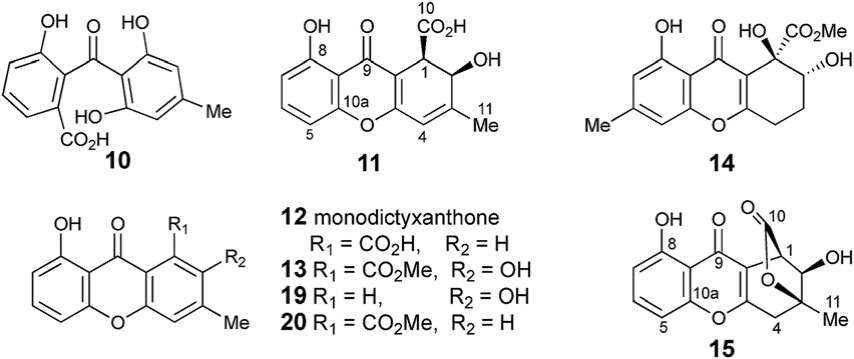
Cephalone F **10** and xanthones discussed in the text.

The methyl signal showed long range couplings and COSY correlations to signals at *δ*_H_ 6.08 and 4.86, the latter being further coupled to a doublet (8.5 Hz) at 4.07 ppm. COSY and HMBC correlations (Fig. 2), were consistent with the dihydroxanthone structure **11**. Slow crystallization from methanol gave crystals which allowed a high quality X-ray crystal structure (see ESI†) to be obtained (Flack parameter –0.01(4)) which confirmed structure **11**, here named agnestin A, and the (1*R*,2*R*) absolute stereochemistry.^19^ It is relatively stable due to the *gauche* relationship of the carboxyl and hydroxyl substituents although slow dehydration does occur to give the known monodictyxanthone **12** 20 whose presence was also detected in older (>25 days) cultures. The related dehydro-compound **13** (Fig. 3) is known from fungal endophytes of *Picea glauca*,^21^ and the similar tetrahydro-xanthone analogue, α-diversonolic ester **14** is known from *Penicillium diversum*.^22^

The peak eluting at 14.3 minutes (typical yields of 5–10 mg L^–1^, Fig. 1) was a further structural isomer with the molecular formula C_15_H_12_O_6_ (HRMS calc. 311.0532, measured 311.0521 for [M + Na]^+^). The ^1^H NMR spectrum indicted a close resemblance to agnestin A **11**: three adjacent mutually coupled aromatic hydrogens were clearly present at *δ*_H_ 6.92, 7.58 and 6.78 ppm. The main difference was the presence of two methine singlets (*δ*_H_ 3.87 and 4.11 ppm) and a closely coupled methylene AB signal centred at 3.15 ppm. The two singlets showed long-range COSY correlations to a methyl group (1.53 ppm), and the C-3 resonance at *δ*_C_ 158.1 ppm in agnestin A **11** was replaced by one at 86.8 ppm. These observations, combined with HMBC correlations (Fig. 2) were consistent with the bicyclic lactone-containing structure **15**, which we have named agnestin B.

Consistent with this, samples of agnestin A **11** were observed (HPLC) to slowly convert to agnestin B **15** and give colourless crystals on slow evaporation from acetonitrile. X-ray analysis (see ESI†) confirmed the structure of agnestin B **15** and also explained the lack of observable coupling between H-1 and H-2 due to the observed dihedral angle of 77° (see ESI†). The 1*R*,2*R* absolute configuration of agnestin B **15** was assigned on the basis of its formation from rearrangement of agnestin A **11**.

To examine if either agnestin A **11** or agnestin B **15** was merely an extraction-artefact formed by acid-catalysed rearrangement of the other, a comparison of extraction conditions was made. One culture was worked-up under the normal acidified conditions, while another was extracted under neutral conditions. In each case both agnestins were observed, suggesting that both are true *in vivo* products.

Extracts of younger cultures of *P. variotii* (4–5 days) show the presence of a major peak at 10.2 minutes with a molecular formula of C_15_H_14_O_7_ (calc. 305.0667, measured 305.0660 for [M – H]^–^) corresponding to formal addition of H_2_O to the agnestins. However, on attempted isolation, the compound rearranged to give mainly agnestin A **11** with some agnestin B **15** (see ESI†). Initial NMR studies of a freeze-dried HPLC-purified fraction showed the presence of two major components, one of which appeared to be a benzophenone due to the presence of the characteristic *ortho* coupled one-proton triplet and two proton doublet associated with the “symmetrical” 2,6-dihydroxyphenolic ring. The other component has the triplet, doublet, doublet pattern more typical of constraining this ring into a xanthone derivative. Other signals are present at *δ*_H_ 5.8, 4.7, 3.8, 3.2 and 2.1 ppm. Similar signals were observed in a spectrum obtained directly from an HPLC-purified fraction (WET ^1^H NMR with solvent suppression)^23^ using a 600 MHz cryo-probed spectrometer. Monitoring the ^1^H NMR spectrum over time showed that the mixture gradually converted to mainly agnestin A **11** after 15 hours (see ESI†). Simple biosynthetic rationale requires the 2–OH to be introduced by an oxidation at C-2 of monodictyphenone **7**, and the molecular formula requires a further reduction to give the dihydroxanthone core.

**Scheme 2:**
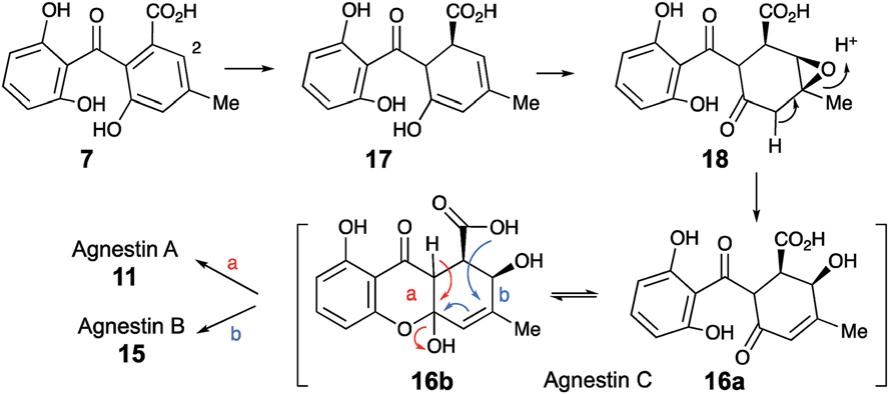
Postulated formation of agnestins A **11** and B **15***via* agnestin C **16ab**.

We propose that this intermediate, agnestin C, is an equilibrating *ca.* 50 : 50 mixture of structures **16a** and **16b** which could be formed *via* epoxidation of the dihydro-derivative **17** of monodictyphenone **7** to give epoxide **18** followed by rearrangement to give the allylic alcohols **16a**/**b**. In the absence of evidence to the contrary it is of course possible that epoxidation could precede reduction. Elimination of water could give agnestin A **11** or agnestin B **15** by either of the mechanisms shown in Scheme 2.

In addition to monodictyxanthone **12**, older (*ca.* 25 days) cultures contained two further minor xanthone derivatives. These were readily identified by comparison of their spectroscopic characteristics with a compound **19** reported^24^ by Tarcz *et al.* and a natural product **20** isolated by Li *et al.* from co-cultures of two South China coast fungal strains (Fig. 3).^25^ The former is presumably formed from agnestin A by decarboxylation and oxidation, whereas the latter corresponds to the methyl ester of monodictyxanthone **12**. Interestingly, these extracts also contained detectable amounts of the anthraquinones emodin **9** and chrysophanol **6**.

## Genetic analysis

A draft genome sequence of *P. variotii* K5103 was previously obtained.^16^ Identification of a putative biosynthetic gene cluster (BGC) responsible for the biosynthesis of the agnestins (*agn*) was aided by comparison with the known *mdp***7** and *ged***8** clusters from *A. nidulans* (Table 1).^9^,^10^

**Table 1:** Analysis of the agnestin biosynthetic gene cluster from *P. variotii* K5013. Proteins homologous with those encoded by the *mdp* and *ged* BGC are shaded. Mdp, monodictyphenone BGC, Ged, geodin BGC, nr-PKS, non-reducing polyketide synthase

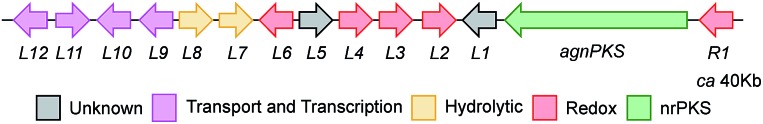					
Gene	Putative function	Swissprot homolog (% id.)	Mdp homolog (% id.)	Ged homolog (% id.)	Ref.
*agnL12*	MFS transporter	lepC, B8NJG7 (39)	—	—	27
*agnL11*	Transcription factor	lepB, B8NJG5 (31)	—	—	—
*agnL10*	Transcription factor	MdpE, Q5BH32 (33)	MdpE, (33)	GedR, (34)	28
*agnL9*	Regulation	MdpA, (43)	MdpA, (43)	GedD, (40)	
*agnL8*	Dehydratase	SCD1, Q00455 (63)	MdpB, (57)	—	29
*agnL7*	Hydrolase	MdpF, Q5BH31 (69)	MdpF, (69)	GedB, (65)	30
*agnL6*	Reductase	CPUR_05429, M1W270 (74)	MpdC, (72)	—	31
*agnL5*	Unknown	PtaG, A0A067XNI6 (43)	—	—	—
*agnL4*	Oxidoreductase	TpcG, Q4WQZ1 (59)	MdpK, (59)	GedF, (61)	32
*agnL3*	BVMO	CPUR_05427, M1WG92 (51)	MdpL, (42)	GedK, (43)	33
*agnL2*	Anthrone oxidase	GedH, P0DOB2 (46)	MdpH2, (43)	GedH, (46)	34
*agnL1*	Decarboxylase	TpcK, Q4WQY7 (72)	MdpH1, (59)	GedI, (67)	
*agnPKS*	nr-PKS	MdpG, Q5BH30 (66)	MdpG, (66)	GedC, (65)	35
*agnR1*	Oxidoreductase	—	—	—	—

The proposed *agn* BGC consists of *agnPKS*, encoding a fungal non-reducing polyketide synthase 66% identical to the MdpG PKS, flanked downstream by *agnL1* to *agnL12* and upstream by *agnR1*. Nine genes are common in the *mdp*, *ged* and *agn* clusters (translated protein identities >42%, Table 1). In addition *agnL10* encodes a transcription factor with 33% identity to the transcription factor MdpE. Four putative ORFs that are specific to the *P. variotii agn* cluster were also identified (*agnR1*, *agnL5*, *agnL11* and *agn L12*, Table 1).^26^

To confirm that we had identified the correct cluster, the *agnPKS* was knocked-out using a bipartite strategy.^36^ LCMS analysis of the *ΔagnPKS* strain, showed complete loss of monodictyphenone **7**, agnestins and all related compounds (see ESI†). Knockout of *agnL3*, which shows 42% identity with *mdpL*, encoding a Baeyer–Villiger oxidase, also caused total loss of monodictyphenone **7** and agnestin biosynthesis. The mutant accumulated emodin **9** and chrysophanol **6** (677 and 791 mg L^–1^ respectively, see ESI†), consistent with the predicted role for AgnL3 in anthraquinone ring cleavage.

AgnL4 shows high homology (59%) with MdpK which had been assigned^10^ a role in rearrangement of an epoxide intermediate in the proposed conversion of emodin **9** to monodictyphenone **7** by analogy to that proposed for AflX during the conversion of versicolorin A **24** to desmethylsterigmatocystin **1**.^2^ However, the appropriateness of this analogy has been questioned by Simpson and it has been suggested instead that MdpK may act as a thiolester reductase during the conversion of the Baeyer–Villiger lactone product of MdpL oxidation of chrysophanol **6** to an aldehyde equivalent of monodictyphenone.^37^

**Scheme 3:**
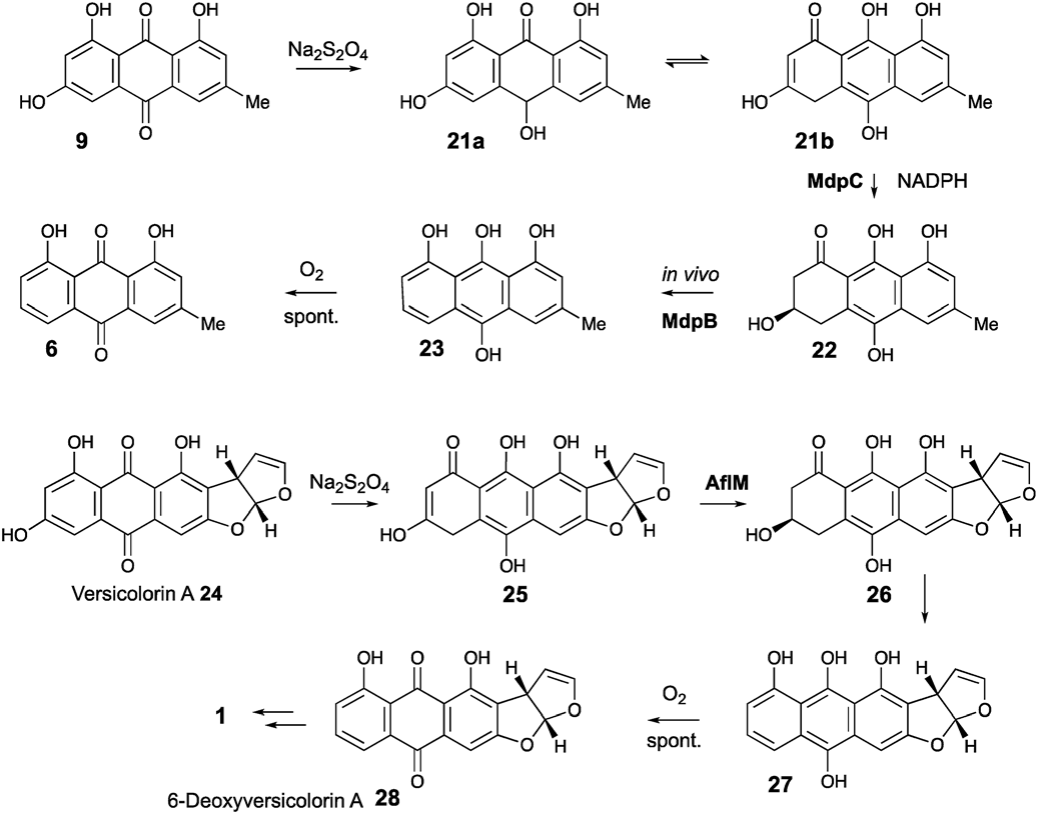
Prior reduction of anthraquinones **9** and **24** is required before phenol reduction.

Our results are not consistent with either of these possibilities. Knock-out of *agnL4* results in accumulation of emodin **9**, but only traces of chrysophanol **6** (see ESI†). Müller has shown^38^ that the initial “ketoreduction” step during the conversion of **9** to **6***in vitro* requires prior chemical reduction of **9** to the hydroquinone (shown by NMR to exist in the tautomeric forms **21a** and **21b**) before MdpC (=AgnL6) mediated reduction to hydroxyketone **22** (and subsequent MdpB [=AgnL8] mediated dehydration to give chrysophanol **6**) can occur *in vitro* (Scheme 3). In a subsequent study with Townsend,^39^ it was shown that AflM (67% amino acid identity to MdpC, = AgnL6), will also reduce emodin **9** and versicolorin A **24** to their corresponding hydroxyketones **22** and **26**, but again only after prior chemical (dithionite) reduction to the corresponding dihydroquinones **25** and **21** respectively (Scheme 3). Thus the most likely *in vivo* role of AgnL4, and by extension MdpK, is to reduce emodin **9** to its hydroquinone **21**, which is the true substrate for AgnL6/MdpC. Likewise, we propose that AflX (43% identity to AgnL4) reduces versicolorin A **24** to its corresponding dihydroquinone **25** before the subsequent AflM-mediated conversion to 6-deoxy-versicolorin A **28** in the sterigmatocystin pathway.

**Scheme 4:**
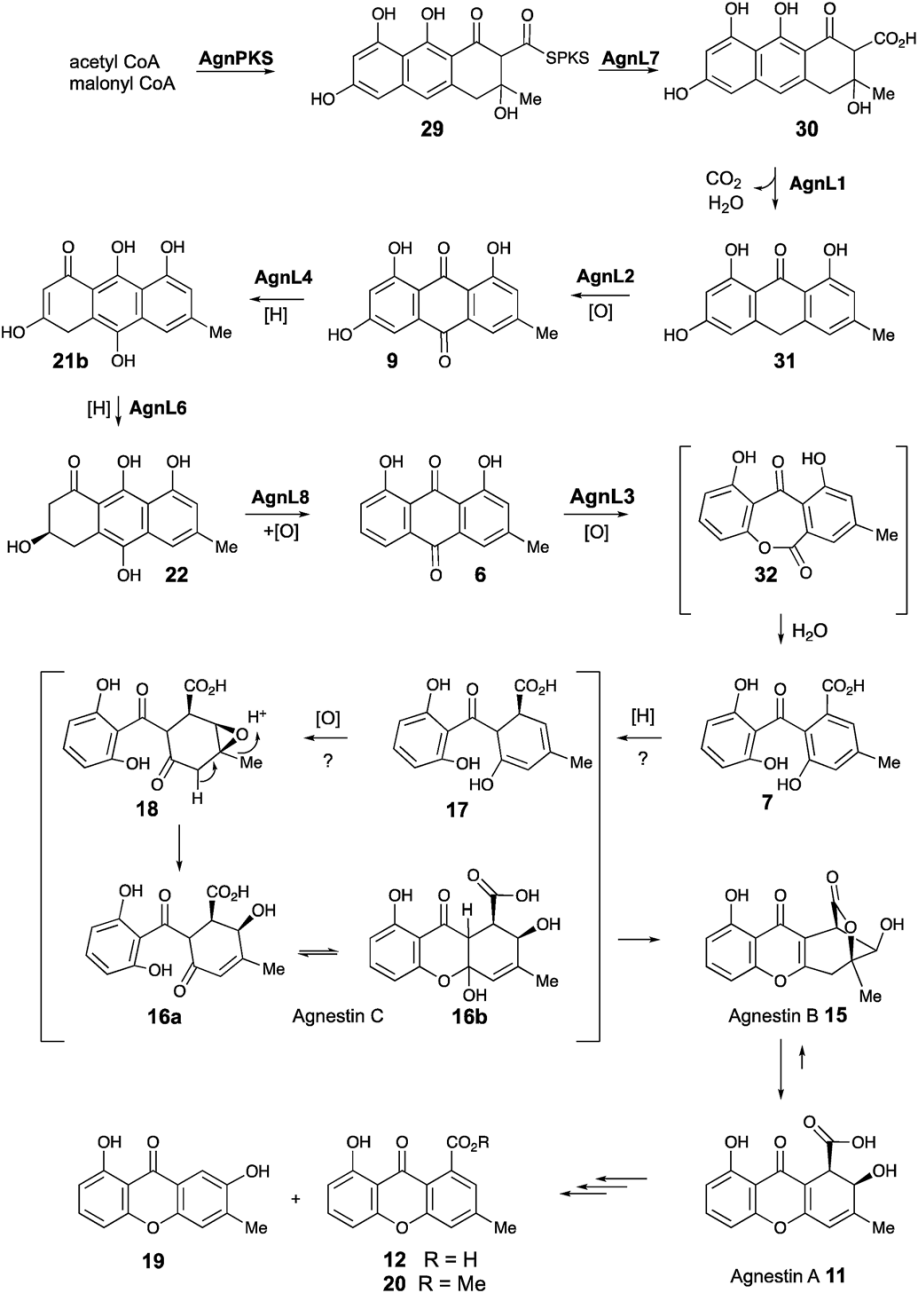
Biosynthesis of agnestins and related phenolic metabolites in *P. variotii*.

Based on these observations we now propose a unifying pathway to monodictyphenone and the agnestins (Scheme 4) which is consistent with all available evidence. AgnPKS (=MdpG) assembles and cyclises an octaketide bound to the PKS as a thiolester. This is then hydrolysed by AgnL7 (=MdpF) to give atochrysone carboxylic acid **30** as the first enzyme-free intermediate. AgnL1 then catalyses the concerted decarboxylation-elimination required to convert atochrysone carboxylic acid **30** to emodin anthrone **31** which is then oxidized to emodin **9** by AgnL2 (=MdpH1). Emodin **9** then undergoes reduction catalysed by AgnL4 (=MdpK) to give the dihydroquinone tautomer **21b**. This is the substrate for AgnL6 (=MdpC) reduction to **22**, followed by AgnL8 (=MdpB) dehydration and likely spontaneous autoxidation to chrysophanol **6**. Baeyer–Villiger oxidation by AgnL3 (=MdpL) gives monodictyphenone **7** along with some cephalone F **10**. Formation of **7** presumably occurs *via* hydrolysis of the lactone **32** which has not been previously reported as a natural product. Close examination of the NMR spectra of isolated samples of monodictyxanthone **12** (see ESI†), show the presence of a co-eluting structural isomer. The chemical shifts are fully consistent with values calculated for **32** which we name monodictylactone.

Further conversion to agnestins A **11** and B **15** (Scheme 4), requires reduction to dihydro-monodictyphenone **17**, oxidation to agnestin C **16ab** probably *via***18**, and rearrangement to either agnestin A **11** or agnestin B **15** directly, although we have demonstrated that **11** and **15** also interconvert. Examination of the *agn* cluster, reveals *agnR1* as the only unassigned oxidoreductase encoding gene present which could be involved in this conversion. KO of *agnR1*, however, revealed it is not involved in the pathway (see ESI†), and thus genes involved in the proposed oxidation/reduction may be located elsewhere on the genome of *P. variotii*. Such split BGCs have been observed before in related systems, for example prenyl transferases involved during the biosynthesis of the shamixanthones in *A. nidulans* are not encoded within the *mdp* BGC itself.^11^ The remaining metabolites **12**, **19** and **20** are probably formed by spontaneous decarboxylations, dehydrations and methanolysis reactions (Scheme 4).

Thus we have identified the protein responsible for the first essential reductive step in the aromatic deoxygenation of anthraquinones, *e.g.* emodin **9** to chrysophanol **6**. The chemical requirement for this had been elegantly demonstrated by Müller and co-workers using an initial chemical reduction step. We have now firmly established the genetic and biochemical basis for this important process.

## Conflicts of interest

There are no conflicts to declare.

## Supplementary Material

Supplementary information

Crystal structure data
