# Supplementary material for: Characterisation of the biosynthetic pathway to agnestins A and B reveals the reductive route to chrysophanol in fungi
Source: Chem Sci. 2018 Nov 26;10(1):233–8. doi: 10.1039/c8sc03778g (PMC6335632; doi:10.1039/c8sc03778g)
Supplement: Supplementary file 1 [file SC-010-C8SC03778G-s001.pdf]

Electronic Supplementary Material (ESI) for Chemical Science.
This journal is © The Royal Society of Chemistry 2018

Characterisation of the Biosynthetic Pathway to Agnestins A and B Reveals the Reductive Route to Chrysophanol in Fungi

Agnieszka J. Szwalbe,[a] Katherine Williams,[a] Zongshu Song,[a] Kate de Mattos-Shipley,[a] Jason L. Vincent,[b] Andrew M. Bailey,[c] Christine L. Willis,[a] Russell J. Cox[a,d,e] and Thomas J. Simpson*[a]

a. School of Chemistry, University of Bristol, Cantock's Close, Bristol, BS8 1TS, UK.

b. Syngenta, Jeallott's Hill, Bracknell, Berkshire, RG42 6EY, UK.

c. School of Biological Sciences, University of Bristol, 24 Tyndall Avenue, Bristol BS8 1TH, UK.

d. Institute for Organic Chemistry, Leibniz University of Hannover, Schneiderberg 1B, 30167, Hannover, Germany.

e. BMWZ, Leibniz Universität Hannover, Schneiderberg 38, 30167, Hannover, Germany.

Electronic Supplementary Information

**1.0**	**Experimental Details**	**2**
1.1	Analytical LCMS	**2**
1.2	Preparative LCMS	**2**
1.3	NMR	**2**
**2.0**	**Characterisation of Compounds**	**3**
2.1	NMR and MS data 7, 10, 11, 12, 15, 16ab, 19, 20, 32	**3**
2.2	Crystal Growth, Solution and Deposition	**20**
2.3	Interconversion of Agnestin A **11** and Agnestin B **15**	**21**
**3.0**	**Sequence Details and Knockout Experiments**	**23**
3.1	Gene cluster details + Bioinformatics	**23**
3.2	Transformation and KO procedure	**26**
3.3	ΔAgnPKS	**28**
3.4	ΔAgnL4	**28**
3.5	ΔAgnL3	**29**
3.6	ΔAgnR1	**31**
**4.0**	**References**	**34**

1.0 Experimental Details

1.1 Analytical LCMS

Samples (20 μL, 10 mg·ml^{-1}) were analysed using a Waters 2795HT HPLC system. Detection was achieved by uv between 200 and 600 nm using a Waters 998 diode array detector, and by simultaneous electrospray (ES) mass spectrometry using a Waters ZQ spectrometer detecting between 150 and 600 *m/z* units. Chromatography (flow rate 1 mL·min^{-1}) was achieved using a Phenomenex Kinetex column (2.6 μ, C_{18}, 100 Å, 4.6 × 100 mm) equipped with a Phenomenex Security Guard precolumn (Luna C_5 300 Å). Solvents were: **A**, HPLC grade H_2O containing 0.05% formic acid; **B**, HPLC grade MeOH containing 0.045% formic acid; and **C**, HPLC grade CH_3CN containing 0.045% formic acid). Gradients were as follows.

Method 1. MeOH: 0 min, 10% **B**; 10 min, 90% **B**; 12 min, 90% **B**; 13min, 10% **B**; 15 min, 10% **B**.

Method 2. CH_3CN: 0 min, 10% **C**; 10 min, 90% **C**; 12 min, 90% **C**; 13min, 10% **C**; 15 min, 10% **C**.

1.2 Preparative LCMS

Compounds were generally purified using a Waters mass or time directed autopurification system compromising Waters 2767 autosampler, Waters 2545 pump system, Phenomenex Kinetex column (5 μ, C_{18}, 100 Å, 250 × 21.20 mm) equipped with Phenomenex Security Guard precolumn (Luna C_5 300 Å) eluted at 16 mL·min^{-1}. Solvents were: **A**, HPLC grade H_2O containing 0.05% formic acid; **B**, HPLC grade MeOH containing 0.045% formic acid; and **C**, HPLC grade CH_3CN containing 0.045% formic acid. The post column flow was split (100:1) with the minority flow made up with HPLC grade MeOH containing formic acid 0.045% to 1 mL·min^{-1} for simultaneous analysis by Waters 2298 diode array detector between 200 and 400 nm; Waters Quattro Micro ESI mass spectrometer in ES+ and ES- modes between 100 *m/z* and 1000 *m/z*; and Waters 2424 ELS detector. Metabolites were collected into glass test tubes. Combined samples were evaporated under N_2 gas, weighed and dissolved in deuterated solvent for NMR analysis.

1.3 NMR

NMR experiments were conducted on the following spectrometers: Varian 400-MR Varian (^{1}H NMR at 400 MHz and ^{13}C NMR at 100 MHz), Varian VNMR S500 spectrometer, (^{1}H NMR at 500 MHz, ^{13}C NMR at 125 MHz), Bruker Avance III HD Cryo ^{13}C-probe, (^{1}H NMR at 500 MHz and ^{13}C NMR at 125 MHz) and Bruker 700 micro-cryo (^{1}H NMR at 700 MHz). Chemical shifts were recorded in parts per million (ppm referenced to the appropriate residual solvent peak) and coupling constant (J) in Hz, reported to the closest 0.5 Hz. Multiplicity is described by the following abbreviations: s, singlet; d, doublet; t, triplet; q, quartet; m, multiplets.

2.0 Characterisation of Compounds

2.1 Characterisation of 6, 7, 9, 10, 11, 12, 15, 16ab, 19, 20, 32

2.1.1 ***Chrysophanol*** **6**:[2] orange crystalline solid; λ_{max} (LCMS) 225, 257, 290 nm; ^{1}H NMR (400 MHz, DMSO-d_6) δ 1.57 (3H, s), 7.10 (1H, m), 7.29 (1H, dd, 8.5, 1.1), 7.66 (1H, m), 7.67 (1H, t, 8.3), 7.82 (1H, dd, 7.5, 1.1), 12.12 (1H, s), 12.02 (1H, s).

2.1.2 Monodictyphenone **7**:[1] orange oil; λ_{max} (LCMS) 211, 277 nm; ESIMS (LCMS) *m/z* 287.5 [M-H]$^-$, 243.6 [M-H-CO_2]$^-$, 575.9 [2M-H]$^-$; ESIMS (LCMS) *m/z* 289.5 [M]H$^+$;

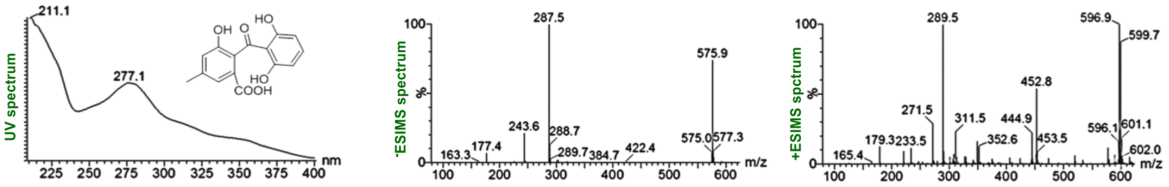

Figure S1. The UV and –ESIMS spectra of monodictyphenone.

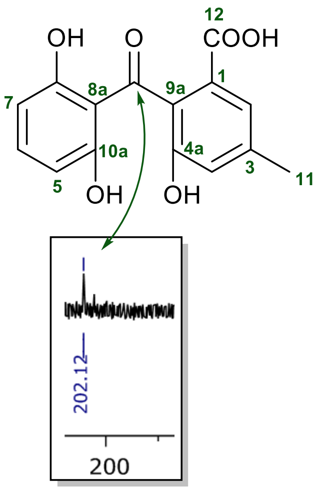

Position	δC Experimental	δC Literature[1]
1	129.5	129.5
2	122.7	122.7
3	139.5	139.5
4	121.1	121.1
4a	154.1	154.1
5	108.0	108.0
6	136.6	136.6
7	108.0	108.0
8	162.9	162.9
8a	112.6	112.6
9*	-*	**202.1**
9a	131.9	131.9
10a	162.9	162.9
11	21.1	21.1

Table S1. Comparison of experimental ^{13}C NMR chemical shifts with the literature data for monodictyphenone (acetone-d_6). * visible in MeCN-d_6 at δ_C 202.1 ppm.

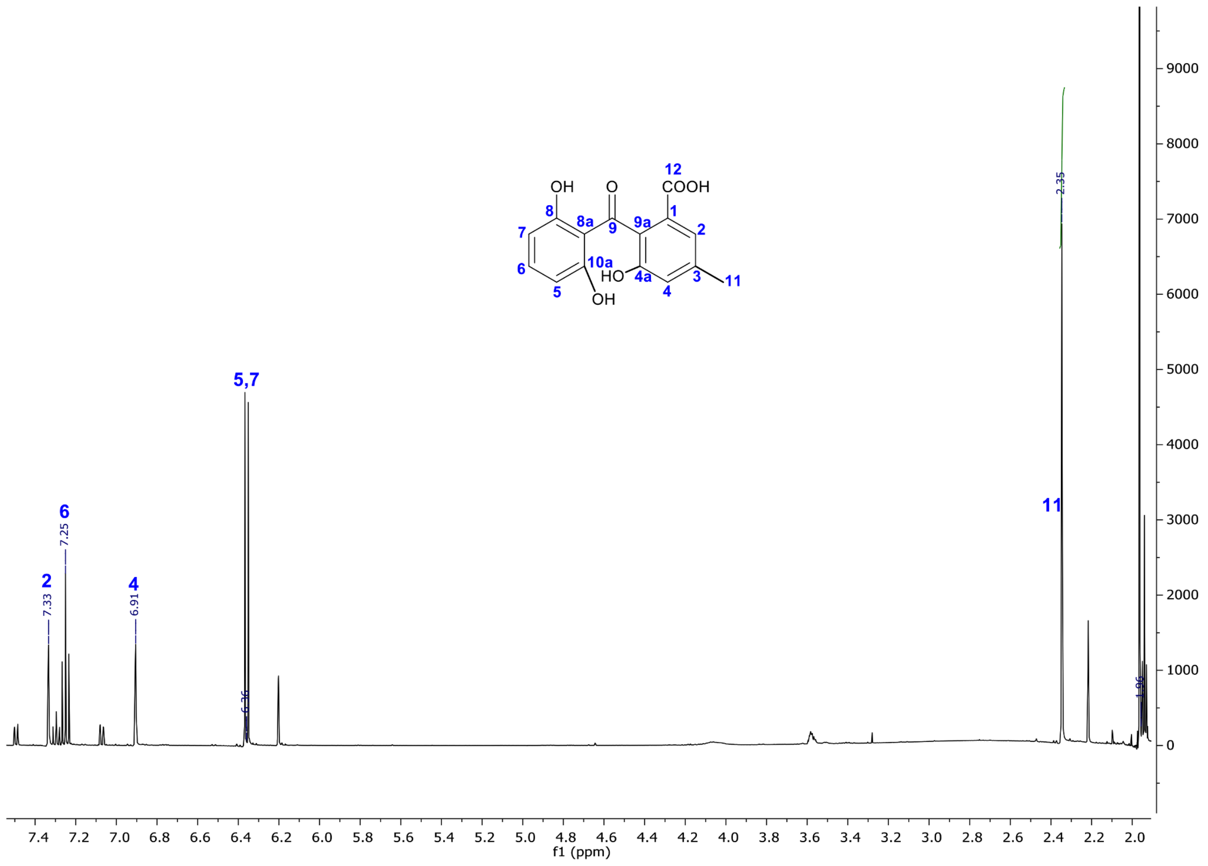

Figure S2. ^{1}H NMR of monodictyphenone **7**

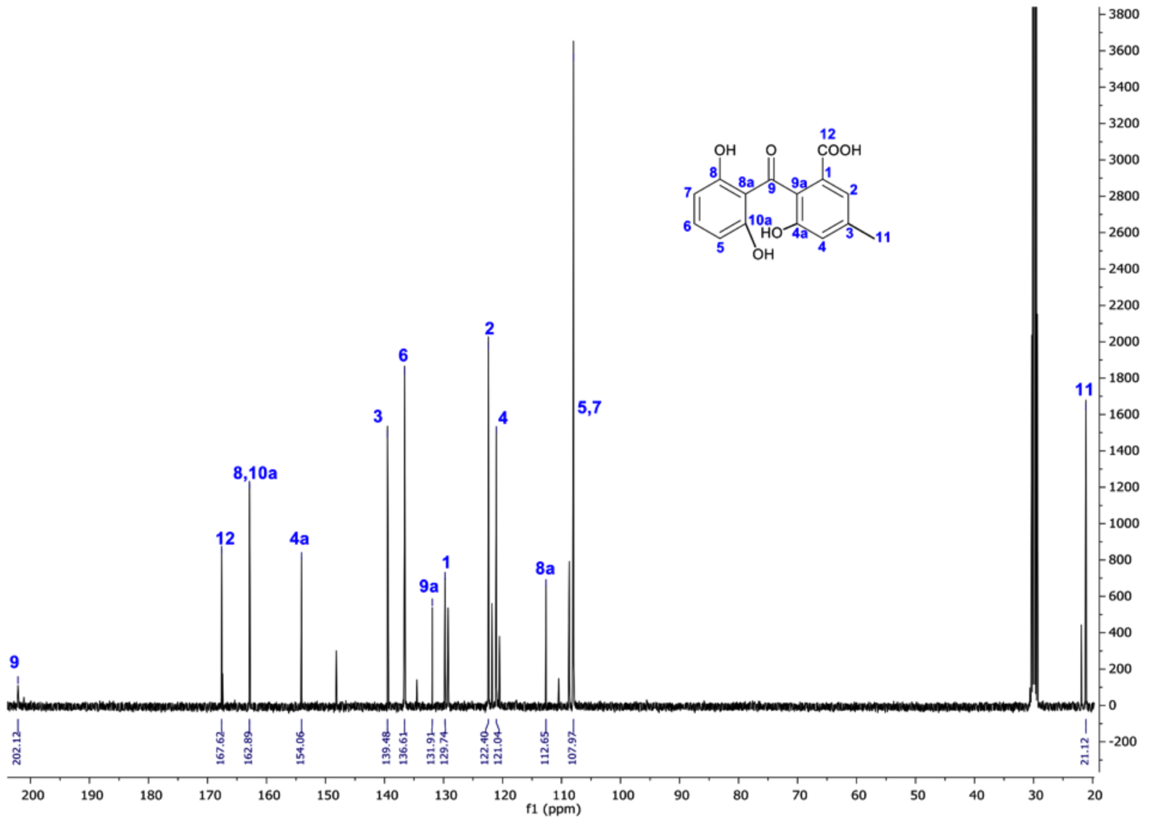

Figure S3. ^{13}C NMR of monodictyphenone **7**

***2.1.3 Emodin* 9**:[2] orange solid; λ_{max} (LCMS) 222, 252, 269, 288 nm; ESIMS (LCMS) *m/z* 269.5 $[M-H]^-$; ESIMS (LCMS) *m/z* 271.5 $[M]H^+$; ^1H NMR: (400 MHz, DMSO-d_6) δ 2.38 (3H, s), 6.55 (1H, d, 2.2) 7.05 (1H, d, 2.4), 7.41 (1H, s), 7.10 (1H, s); ^{13}C NMR (126 MHz, DMSO-d_6) δ 189.4, 181.0, 165.5, 164.3, 161.2, 148.0, 134.8, 132.5, 123.88, 120.2, 113.1, 108.6, 107.7, 21.3.

2.1.4 Cephalone F 10[3]

Close inspection of the ^1H NMR spectrum of monodictyphenone **7** (Fig S4) revealed the presence of its co-eluting structural isomer, cephalanone F **10**.[3]

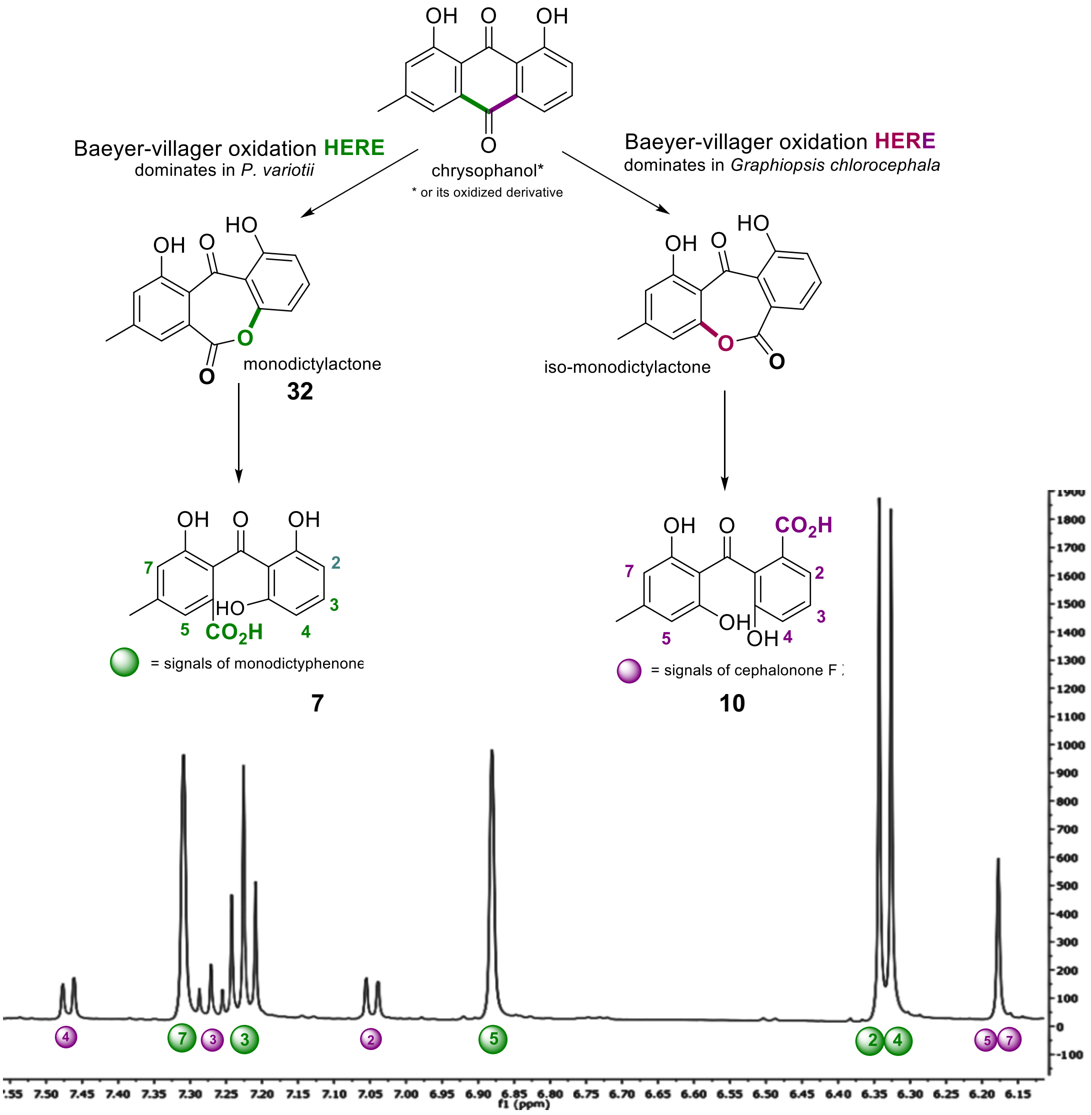

Figure S4. Aromatic region of ^1H NMR spectrum of monodictyphenone **7**, showing minor peaks of co-eluting cephalanone F **10**.

2.1.5 Agnestin A 11: isolated as golden crystals; λ_{max} (LCMS) 220, 270, 358 nm; ESIMS (LCMS) *m/z* 287.5 $[M-H]^-$, 225.5 $[M-H-CO_2-H_2O]^-$, 575.8 $[2M-H]^-$; HRESIMS *m/z* 311.0536 $[M]Na^+$ ($C_{15}H_{12}NaO_6$ requires 311.0532).

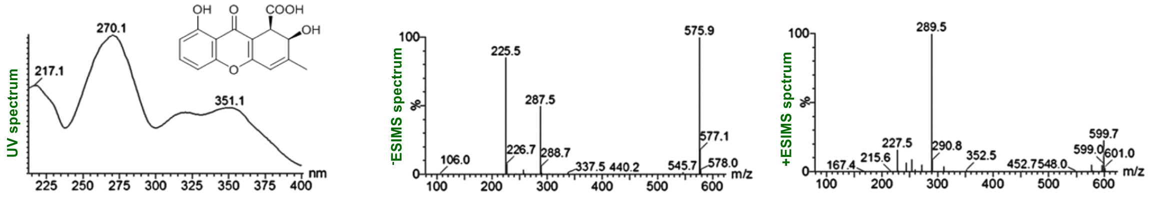

Figure S5. The UV and ESIMS spectra of agnestin A.

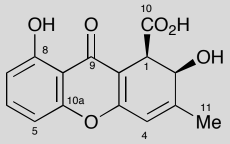

Agnestin A 11

Position	δ_H (*J* / Hz)	δ_C	HMBC
**1**	4.07, d (8.5)	42.5	2, 3, 4, 4a, 9a, 12
**2**	4.86, d*	70.1	1, 3, 4, 4a, 9a, 12
**3**	-	158.1	-
**4**	6.08, s	116.8	2, 3, 4a, 9, 9a, 11
**4a**	-	162.2	-
**5**	6.91, d (8.4)	108.2	7, 8, 9, 10a
**6**	7.55, t (8.3)	136.3	8, 8, 10a
**7**	6.75, d (8.2)	112.0	5, 8, 8a, 9, 10a
**8**	-	161.4	-
**8a**	-	111.2	-
**9**	-	181.7	-
**9a**	-	111.6	-
**10a**	-	156.5	-
**11**	2.05, t (1.6)	19.8	1, 2, 3, 4
**12**	-	172.2	-
**8-OH**	12.57, s	-	7, 8

Table S2. NMR data for **11**.

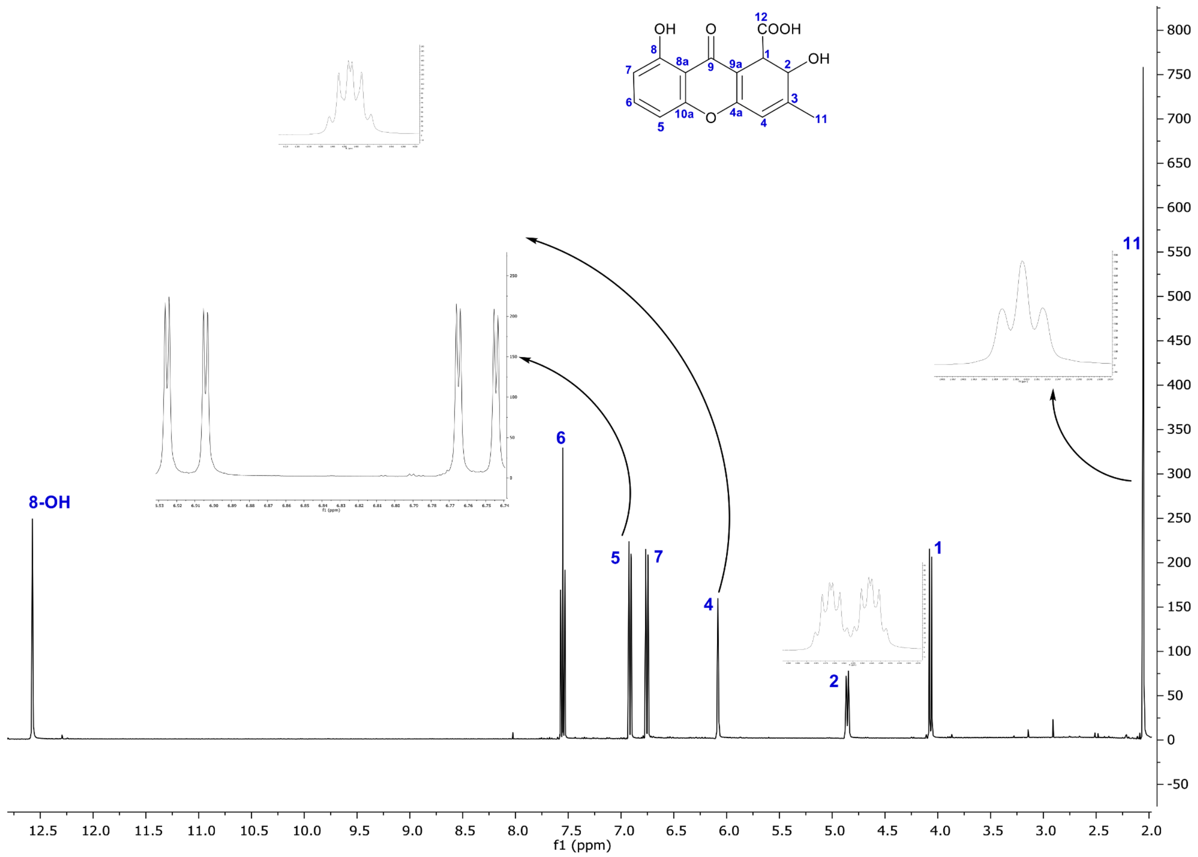

Figure S6. ^{1}H NMR of agnestin A **11** (MeCN-d$_3$).

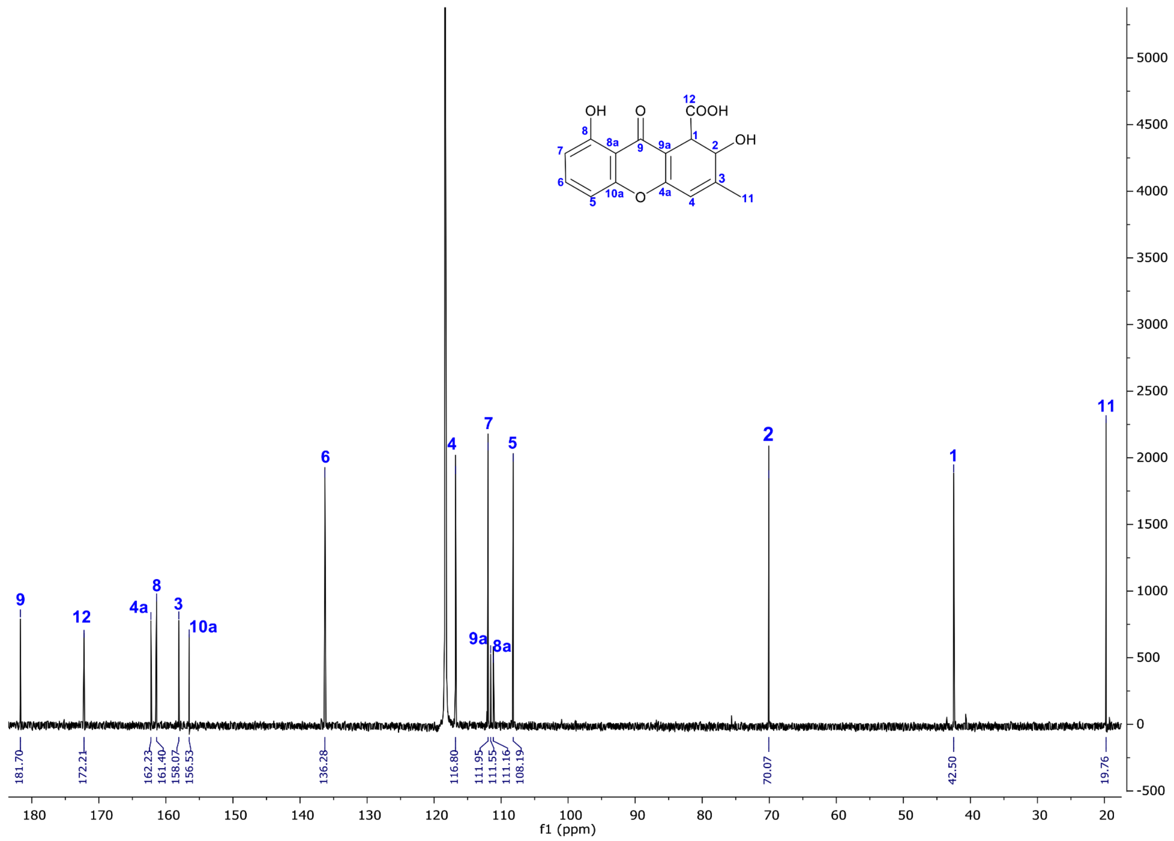

Figure S7. ^{13}C NMR of agnestin A **11** (MeCN-d$_3$).

2.1.6 Monodictyxanthone 12[4] and Monodictylactone 32.

A compound accumulated in older extracts (3 weeks) which eluted at 17.9 min and had a UV spectrum with four maxima (λ_{max} 232, 255, 291, 361 nm), which indicated it to be a xanthone-related compound. LCMS analysis indicated a nominal mass 270 (*m/z* 269.5 in negative ESIMS, *m/z* 271.5 in positive ESIMS, Fig S2.1.6A). Additionally, the LCMS characteristics (t_R, UV and ESIMS spectra) of this compound were exactly the same as for decomposition products observed in older samples of agnestin B **15** and monodictyphenone **7** (**Figure S8**). Furthermore, when old samples of **11** and **7** were mixed and run on the LCMS, a single peak was observed for peak at 17.9 min (**Figure S9**), supporting the idea that the compound was present in both samples.

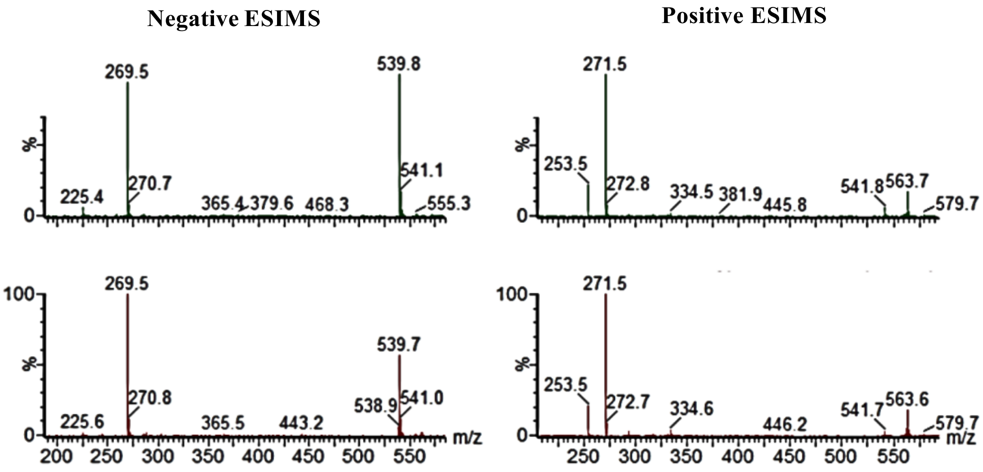

Figure S8. ESIMS spectra of compound with tR 17.9 min in crude extract (top) compared with spectrum of compound with the same tR from a very old sample of agnestin A (bottom)

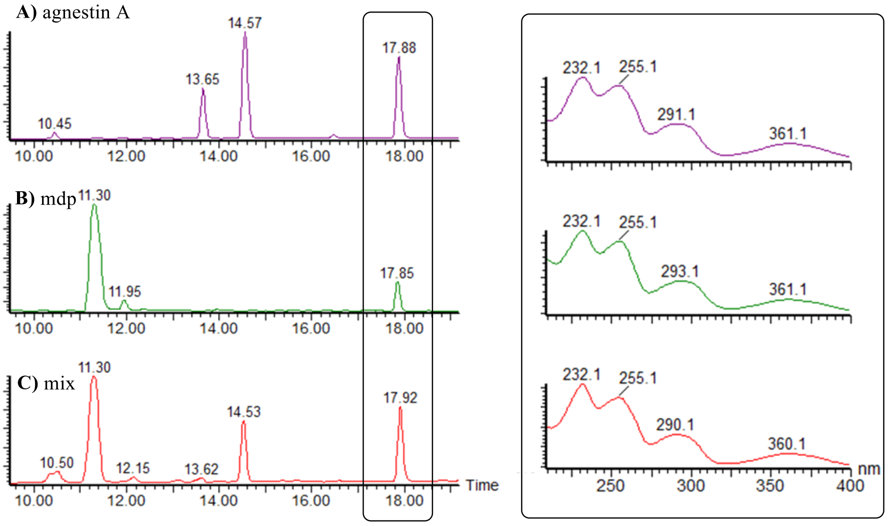

Figure S9. Comparison of Diode Array chromatograms (left) of: A) – old agnestin A sample; B) – old monodictyphenone sample; C) – mixed samples A and B. UV spectra corresponding to peak at tR 17.9 min are shown on the right.

At first glance, all seemed to point to monodictyxanthone **12** being the compound eluting at t_R 17.9 min in crude extracts, which could be formed from both agnestin A **11** and B **15** and monodictyphenone **7**. However, there were no reports that monodictyphenone **7** can undergo spontaneous 4a, 10a - ring-closure to give monodictyxanthone **12**. However formation of lactone **32** (which we name monodictylactone) seems feasible. Monodictylactone **32** and monodictyxanthone **12** are structural isomers that could very well co-elute and were also expected to give practically the same NMR spectra (splitting pattern and HMBC correlations).

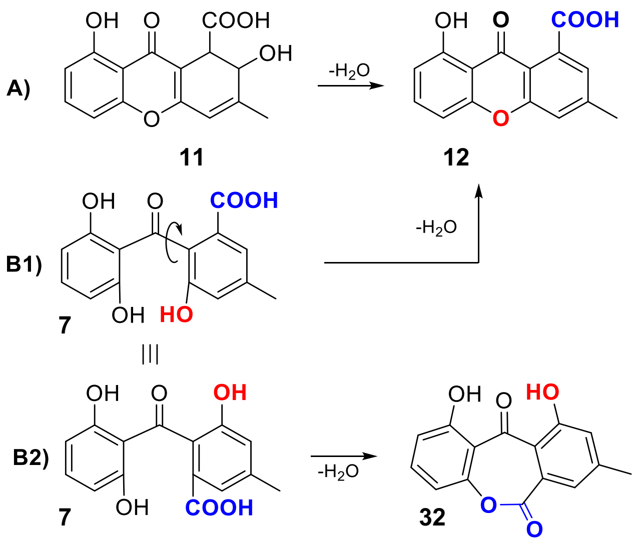

Figure S10. A) Monodictyxanthone **12** was the only possible dehydration product of agnestin A **11** (or B **15**); **B**) - loss of water from monodictyphenone **7** could lead to two isomers: monodictyxanthone **12** or monodictylactone **32**.

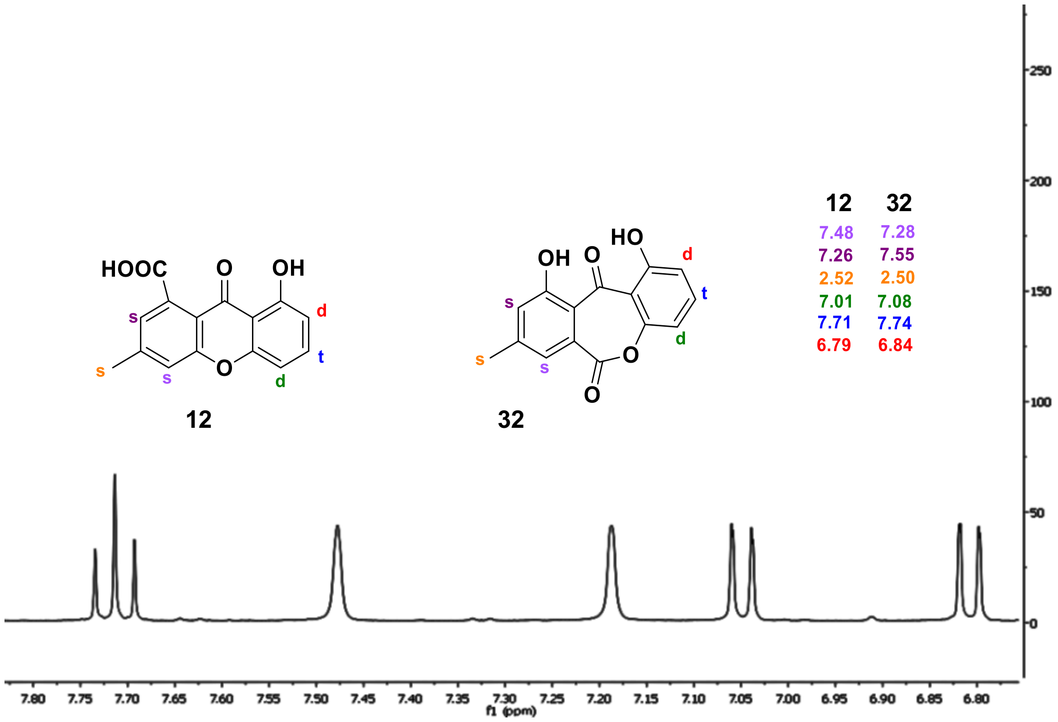

Figure S11. H NMR spectrum of compound purified from the crude extract with calculated ^{1}H NMR shifts for each structure. The NMR data is consistent with either possible structure.

Analysis of NMR spectra of this type of compound is further complicated by keto-enol equilibrium and concentration-dependent chemical shifts. This ambiguity prompted us to reinvestigate compounds formed in the dehydration process occurring in pure samples of monodictyphenone **7** and agnestin B **15** separately.

Aged samples of purified monodictyphenone **7** and agnestin A **11** were examined by LCMS. Both samples contained a degradation peak eluting at 17.9 minutes (Figure S12).

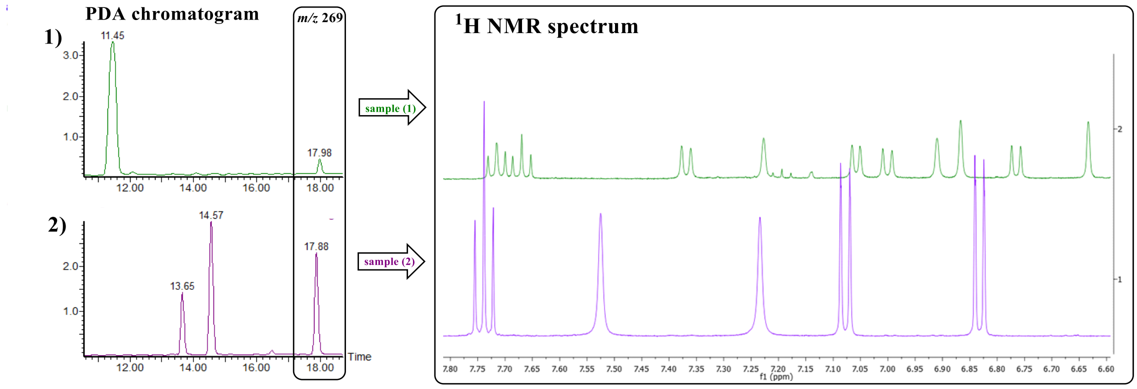

Figure S12. Comparison of the ^{1}H NMR spectrum of compound with tR 17.9 min purified from an old sample of: (1) – monodictyphenone **7**; (2) agnestin A **11**.

As expected, the ^{1}H NMR spectrum of the degradation compound isolated from agnestin A **11** (Figure S2.1.6E) showed pure monodictyxanthone **12**, which was used as a point of reference. The ^{1}H NMR spectrum of degradation product isolated from the monodictyphenone **7** sample (Figure S11)

however, showed two sets of peaks (with ~ 1:1 intensity ratio) with the same splitting pattern as monodictyxanthone **12**, none of which seemed to match the first spectrum. The two samples were then mixed and re-submitted for ^{1}H NMR analysis. The resulting ^{1}H NMR spectrum (Figure S13) showed only two sets of peaks, confirming that monodictyxanthone was indeed one of the degradation components of monodictyphenone **7**. The second component was consequently deduced to be a co-eluting structural isomer - monodictylactone **32**.

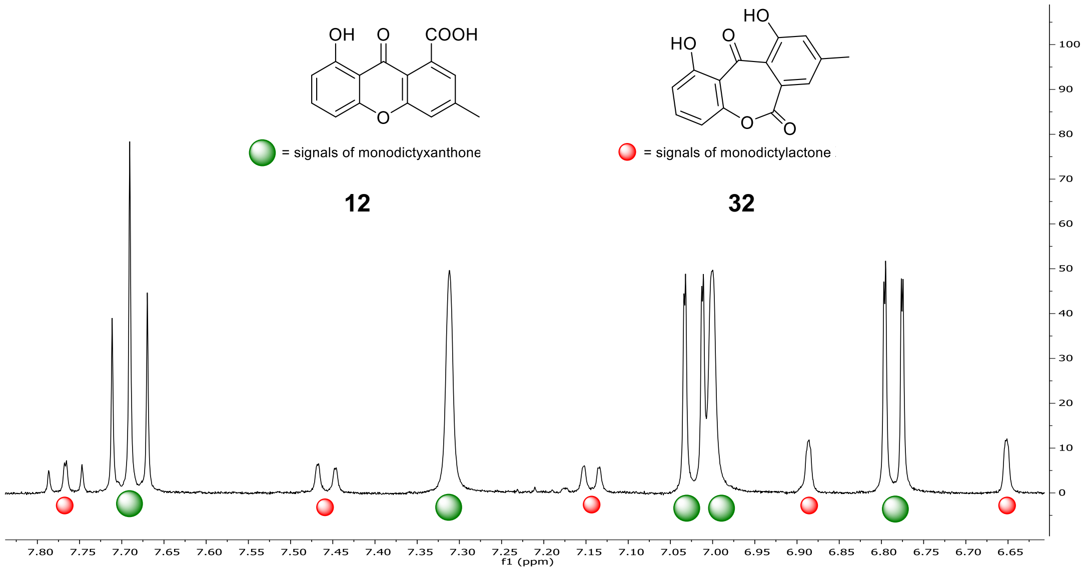

Figure S13. Aromatic region of the ^{1}H NMR spectrum of a mixture of degradation products obtained from aged samples of **7** and **11** . Monodictyxanthone **12** (green balls) and monodictylactone **32** (red balls).

As a result of this detailed analysis, we have established that monodictyphenone **7** does spontaneously close to both **12** and **32** (in ~1:1 ratio based on the relative intensities of signals in the ^{1}H NMR spectrum, Figure S11), although the equilibrium favours the ring-open form.

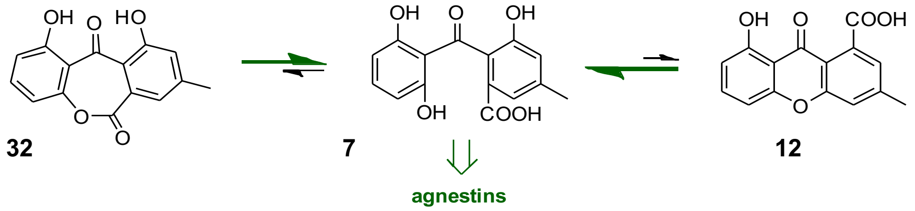

Figure S14. Formation of monodictyphenone **7** appears to be favoured in both reactions shown.

Monodictyxanthone **12**:[4] brownish oil; λ_{max} (LCMS) 232, 255, 291, 361 nm; ESIMS (LCMS) *m/z* 269.5 $[M-H]^-$, 253.5 $[M-H-H_2O]^-$; 539.8 $[2M-H]^-$; ESIMS (LCMS) *m/z* 271.5 $[M]H^+$.

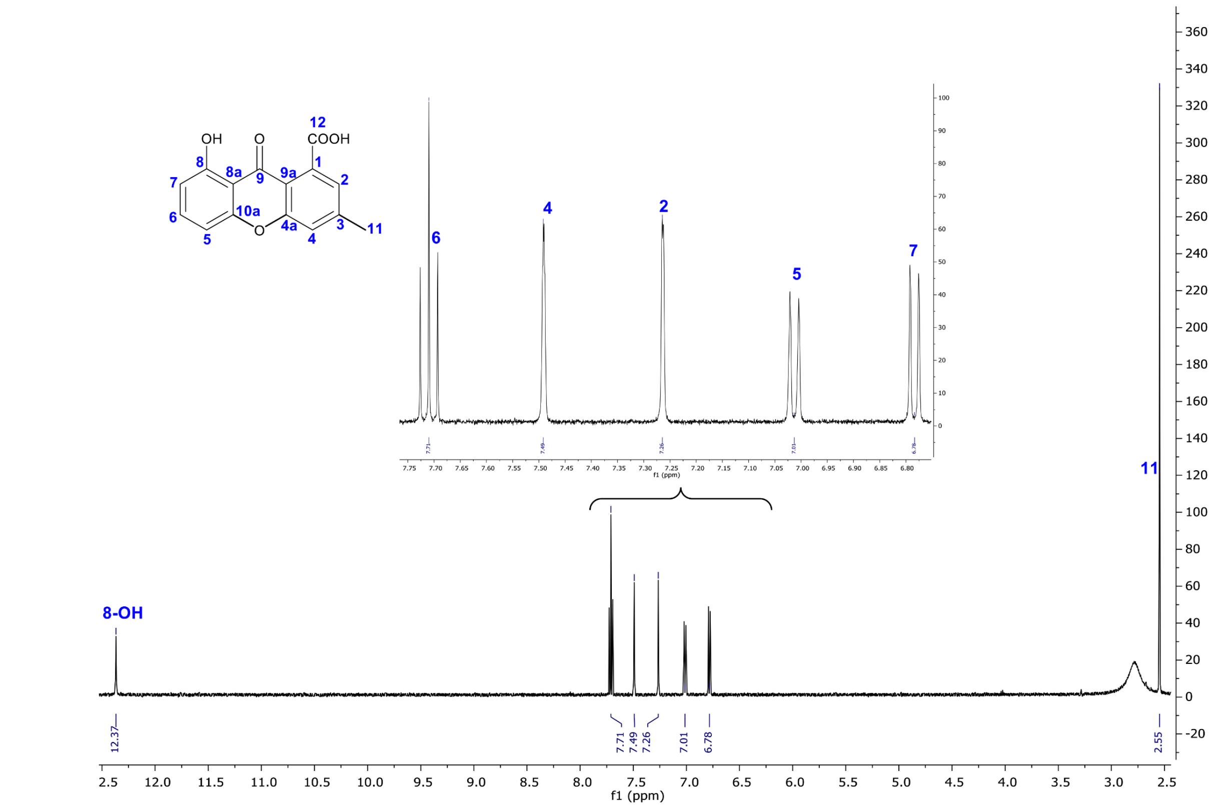

Figure S15. ^{1}H NMR of monodictyxanthone **12**

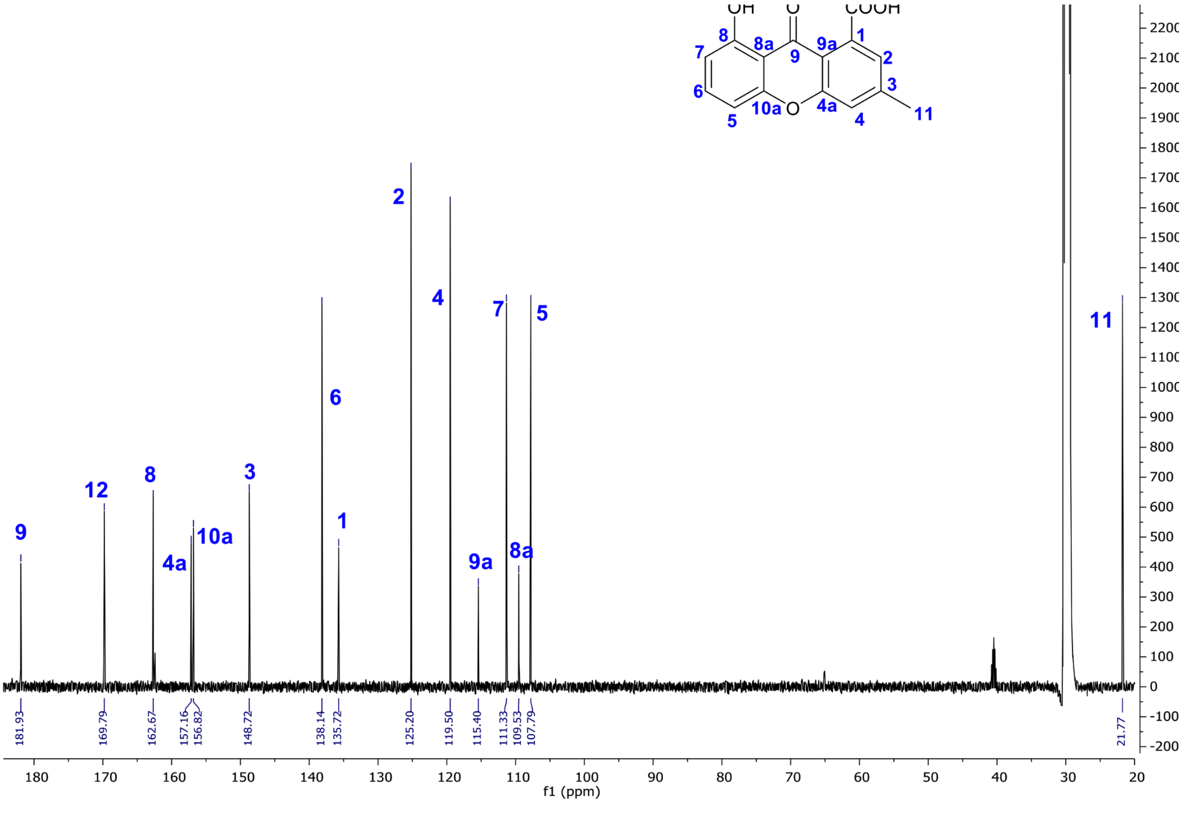

Figure S16. ^{13}C NMR of monodictyxanthone **12**

2.1.7 Agnestin B 15 – isolated as yellow oil; λ_{max} (LCMS) 211, 262, 358 nm; ESIMS (LCMS) *m/z* 287.5 $[M\text{-}H]^-$, 225.5 $[M\text{-}H\text{-}CO_2\text{-}H_2O]^-$, 575.8 $[2M\text{-}H]^-$; HRESIMS *m/z* 311.0521 $[M]Na^+$ ($C_{15}H_{12}NaO_6$ requires 311.0532).

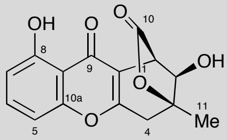

Agnestin B **15**			
**Position**	δ_H (*J* in Hz)	δ_C	HMBC
**1**	3.87, s	43.5	2, 3, 4a, 9, 9a, 12
**2**	4.11, s	75.6	3, 9a, 12
**3**	-	86.8	-
**4**	3.15, d (1.4)	40.7	2, 3, 4a, 9a, 11, 12
**4a**	-	164.0	-
**5**	6.92, d (8.4)	108.2	8a, 7, 10a
**6**	7.58, t (8.4)	136.8	8, 10a
**7**	6.78, d (8.2)	112.1	5, 8a
**8**	-	161.6	-
**8a**	-	111.0	-
**9**	-	180.4	-
**9a**	-	114.7	-
**10a**	-	157.4	-
**11**	1.53, s	19.3	2, 3, 4, 4a
**12**	-	175.1	-
**8-OH**	12.30, s	-	7, 8, 8a

Table S3. ^1H, ^{13}C and HMBC NMR data for **15.**

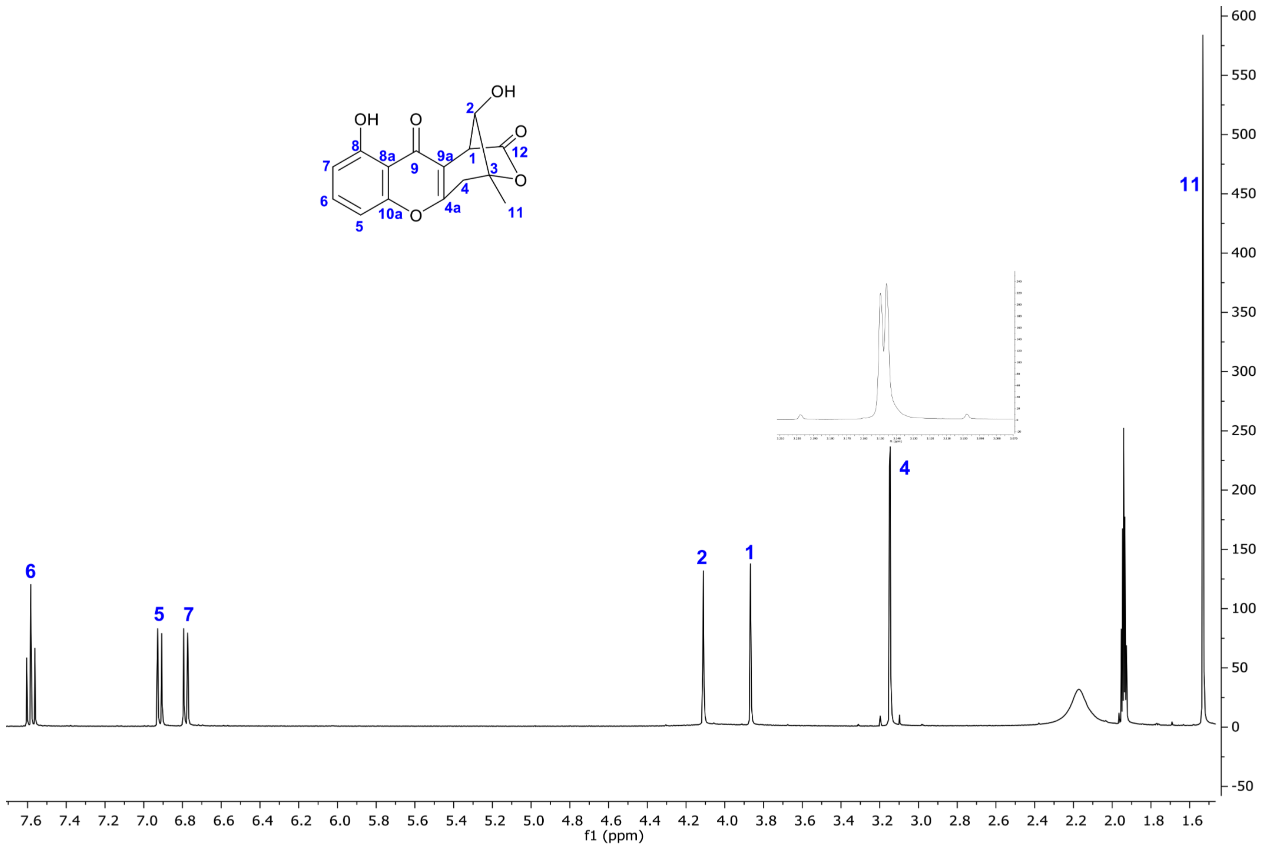

Figure S17. ^{1}H NMR of agnestin B **15** (MeCN-d$_3$)

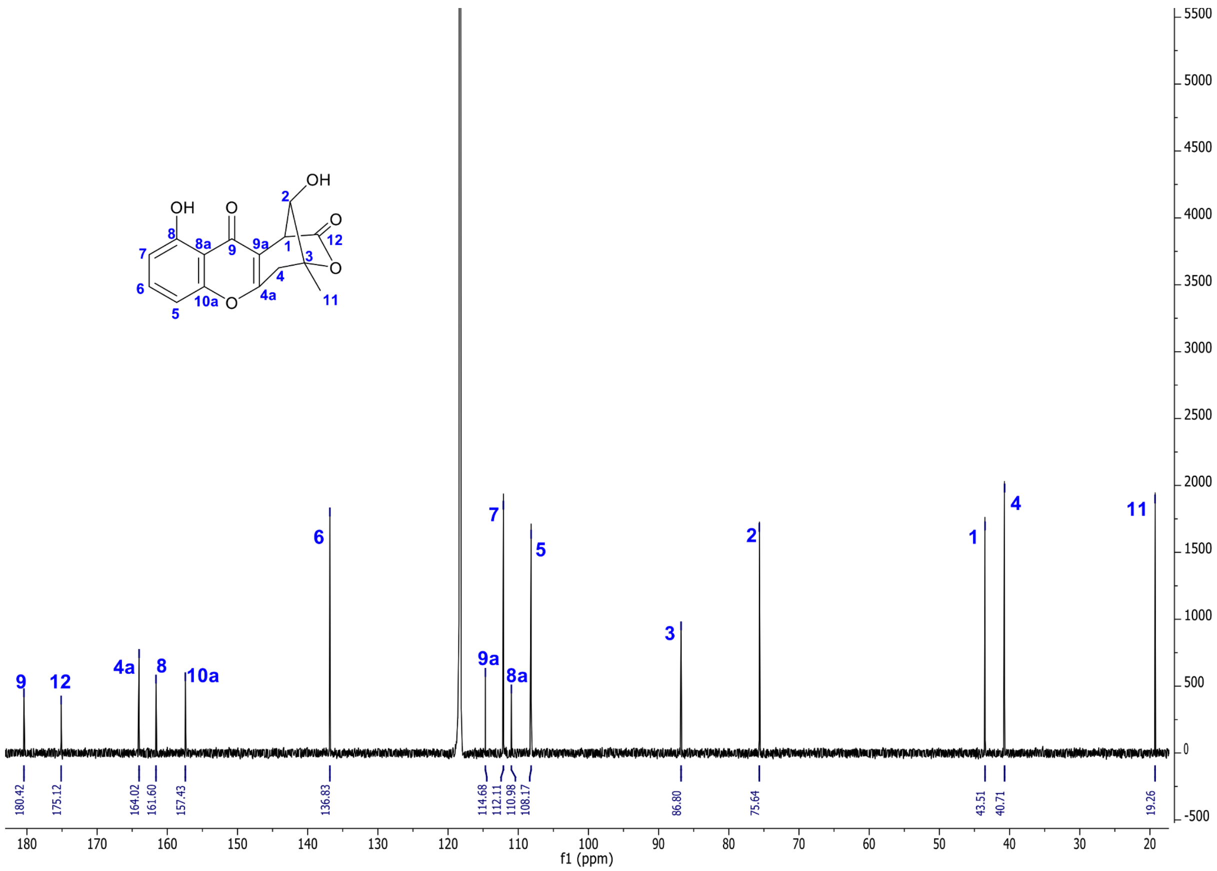

Figure S18. ^{13}C NMR of agnestin B **15** (MeCN-d$_3$)

2.1.8 Agnestin C 16ab

In the younger cultures of *P. variotii* (4-5 days), a metabolite accumulated at t_R 10.4 min. The concentration of this compound fell as the fermentation time increased, later shown to be due to its high instability. The compound had an agnestin A-like UV spectrum and ESIMS spectra (*m/z* 305.6 $[M-H]^-$ in $^-$ESIMS; *m/z* 307.5 $[M+H]^+$ in $^+$ESIMS) which indicated the nominal mass to be 306 (Figure S19) corresponding to a molecular formula of $C_{15}H_{14}O_7$, and indicating formal addion of water to the agnestin A/B structure. The 10.4 min was purified by mass-directed HPLC fractionation. However, the compound was found to rapidly decompose to agnestins A **11** and B **15** (Figure S20) over the period of concentration.

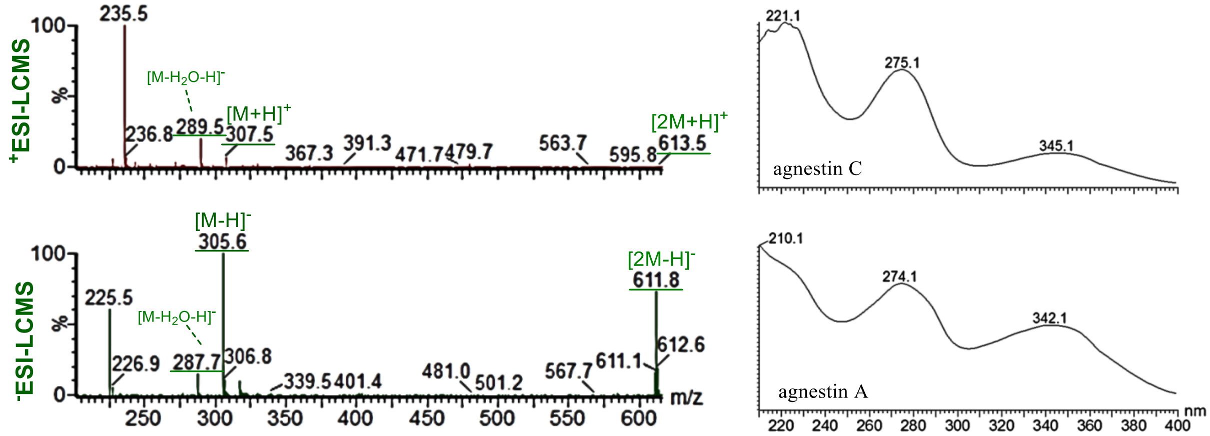

Figure S19. ESIMS spectra of agnestin C **16** (left) and its UV spectrum compared with UV spectrum of agnestin A **11** (right).

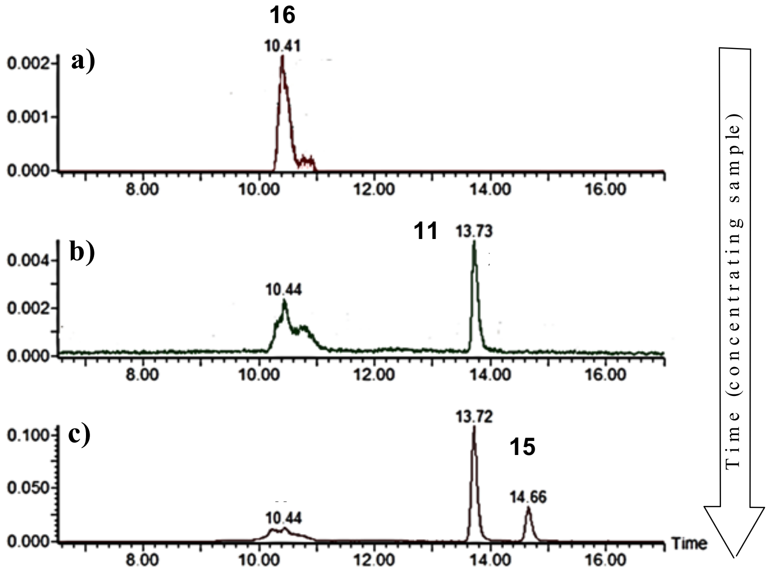

Figure S20. ELSD chromatograms of: **a)** freshly isolated HPLC fractions (t_R 10.4 min), **b)** the same sample concentrated overnight (agnestin A t_R 13.7 min appeared); **c)** the same sample after being dried and re-suspended in MeCN (agnestin B *t* 14.6 min appeared).

Compound **16** was purified by a single HPLC-run of a concentrated extract. The collected fraction was immediately freeze-dried to remove solvent and formic acid, and the residue rapidly analysed by 600 MHz ^{1}H NMR

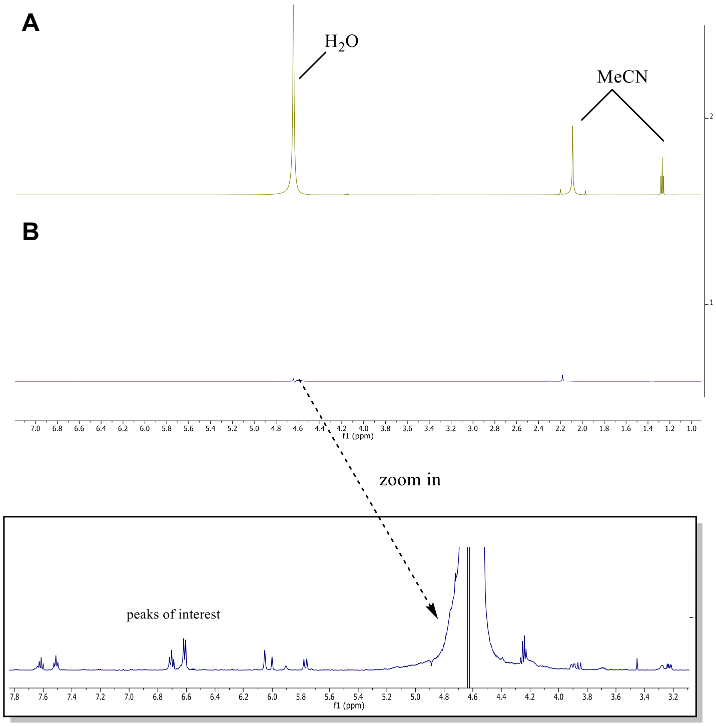

Figure S21. NMR spectra of HPLC fraction containing agnestin C. **A** – ^{1}H NMR spectrum; **B** – WET1D ^{1}H NMR spectrum with solvent suppression.

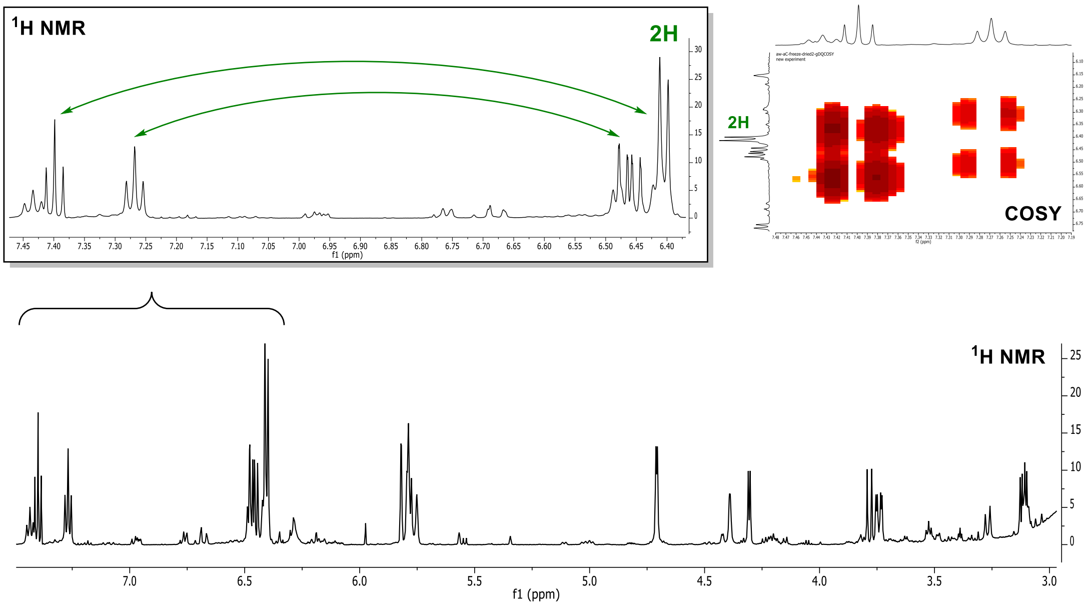

Figure S22. ^{1}H NMR spectrum of freeze-dried HPLC fraction of agnestin C. COSY correlations shown with green arrows.

We also attempted to obtain ^{13}C, COSY, HSQC and HMBC spectra, and although not much structural information could be extracted from the data due to advancing degradation, some important

resonances were observed. One of the compounds appeared to be a benzophenone derivative: there was a doublet of 2H (based on signal integration) showing COSY correlation to one of the two aromatic triplets at δ_H 7.26 (Figure S22), indicating two equivalent protons coupled to the same proton, as in the structure of monodictyphenone (5-H and 7-H appearing as one doublet coupled to 6-H triplet). This indicates that monodictyphenone is the likely precursor for oxidation (Scheme S1). The advancing decomposition was captured by ^{1}H NMR over a period of 15h (Figure S23).

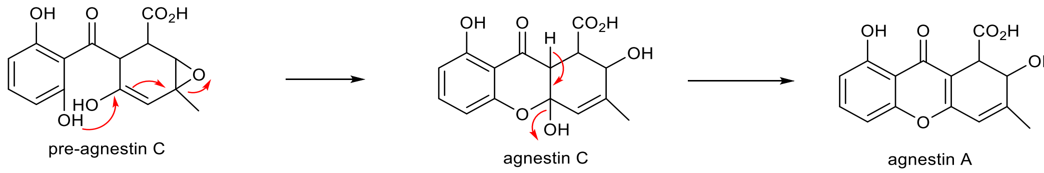

Scheme S1. Possible structures of precursors to agnestin A: pre-agnestin C and agnestin C.

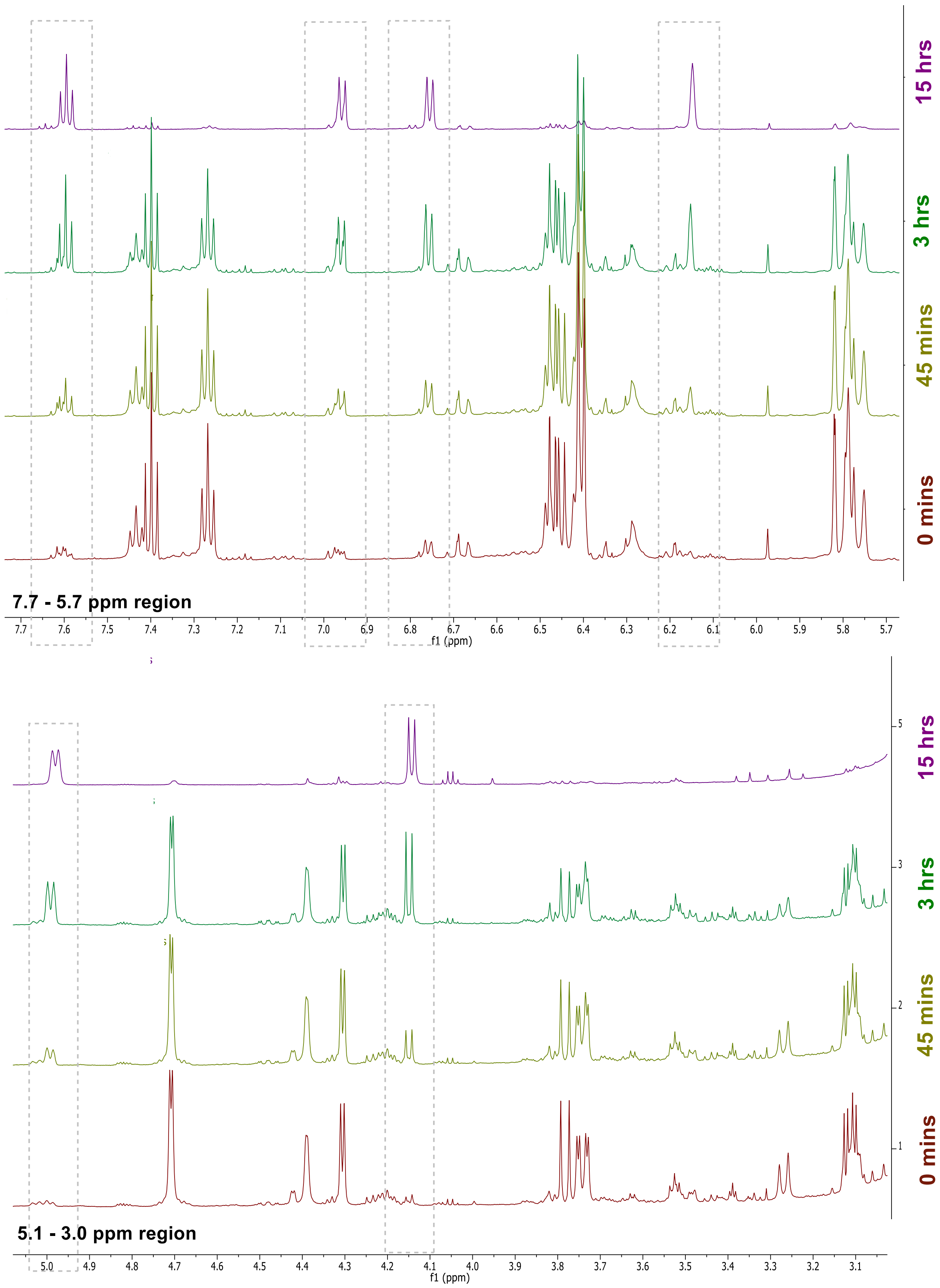

Figure S23. The ^{1}H NMR time-course showing advancing decomposition of isolated Agnestin C **16** to agnestin A **11** (peaks in dotted line boxes.

2.1.9 1,7-dihydroxy-6-methylxanthone 19:[5] yellow oil; λ_{max} (LCMS) 201, 132, 255, 287, 359 nm; ESIMS (LCMS) *m/z* 285.4[M]H$^+$.

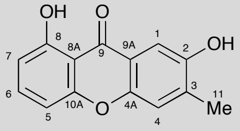

1,7-dihydroxy-6-methylxanthone 19			
**Position**	δ_H *J* / Hz	δ_C	HMBC
**1**	7.58, s	107.1	1, 2, 3, 4, 4a
**2**	-	152.7	-
**3**	-	136.9	-
**4**	7.41, s	119.4	1, 2, 4a, 9, 9a
**4a**	-	150.2	-
**5**	6.98, dd (8.4, 0.7)	106.8	7, 8, 8a, 9, 10a
**6**	7.67, t (8.4)	136.6	8, 8a, 10a
**7**	6.75, d (8.3, 0.7)	109.5	5, 8, 8a, 9, 10a,
**8**	-	161.8	-
**8a**	-	108.2	-
**9**	-	181.8	-
**9a**	-	118.9	-
**10a**	-	156.4	-
**11**	2.40, s	16.2	1, 2, 3, 4
**12**	-	-	-
**8-OH**	12.81, s	-	

Table S4. NMR data for **19**.

2.1.10 8-hydroxy-3-methyl-9-oxo-9H-xanthene-1-carboxylic acid methyl ester 20:[6] yellow oil; λ_{max} (LCMS) 201, 235, 247, 258, 289, 284 nm; ESIMS (LCMS) *m/z* 241.2 [M-H]$^-$; +ESIMS (LCMS) *m/z* 243.2 [M]H$^+$.

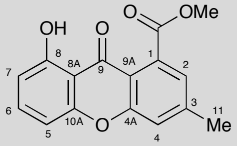

**8-hydroxy-3-methyl-9-oxo-9H-xanthene-1-carboxylic acid methyl ester 20**		
**Position**	δ_H J / Hz	δ_C
**1**	-	133.5
**2**	7.15	124.3
**3**	-	147.1
**4**	7.35	119.3
**4a**	-	156.3
**5**	6.91	107.0
**6**	7.59	137.0
**7**	6.80	111.0
**8**	-	161.9
**8a**	-	109.0
**9**	-	181.0
**9a**	-	115.4
**10a**	-	156.0
**11**	2.52	22.1
**12**	-	169.8
**8-OH**	12.30	-
**12-OCH$_3$**	4.02	53.3

Table S5. NMR data for **20.**

2.2 Crystal Growth, Solution and Deposition

The natural tendency for crystallization of Agnestin A **11** was utilized to obtain a crystal structure by X-ray analysis, which confirmed the structure elucidated by NMR. Tiny crystals left in a vial after evaporation of HPLC solvents were re-dissolved in methanol. Slow evaporation under a nitrogen flow yielded shiny, golden crystals. Crystallographic analysis also determined the absolute stereochemistry (Flack parameter -0.01(4)) of agnestin A 210 to be 1-(*R*), 2-(*R*).

Unlike agnestin A **11**, agnestin B **15** did not readily form crystals during evaporation of HPLC solvents, however, when agnestin B **15** was re-purified from the old samples of agnestin A **11** (where it appeared as decomposition product) colourless crystal could be obtained by slow evaporation of aqueous acetonitrile. The X-ray analysis revealed that agnestin B **15** indeed had the structure as

elucidated by NMR. The three-dimensional structure also provided an explanation as to why adjacent protons H-1 and H-2 appear as singlets in the ^{1}H NMR spectrum, and show no vicinal coupling in the COSY spectrum. A dihedral angle φ1-2 between these protons had a value -77.5°, which in accordance with the Karplus equation corresponds to ^{3}J values being very small or close to zero (equal zero for 90°), and therefore not discernible in the NMR.

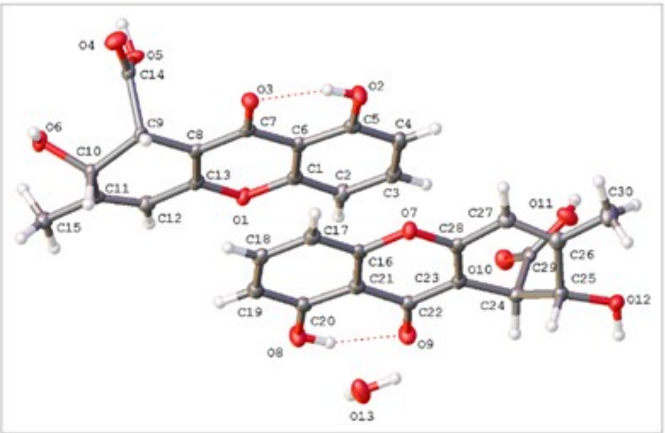

Figure S24. Crystal structure of agnestin A

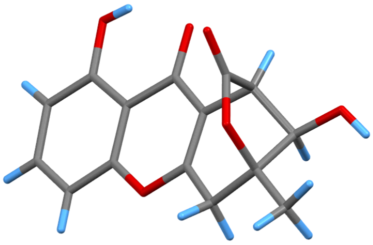

Figure S25. Crystal structure of agnestin B

Crystallographic data hasve been deposited with CCDC: acession numbers1839028-1839029

2.3 Interconversion of Agnestin A 11 and Agnestin B 15

Agnestin A **11** and B **15** were found to interconvert. Agnestin B **15** was observed to undergo rearrangement to agnestin A **11** and the dehydration product - monodictyxanthone **12**. This process occurred spontaneously with time and was catalyzed by acid (Figure S26). A sample of freshly purified agnestin B **15** was acidified and then subjected to LC-MS analysis showing clear conversion to Agnestin A **11** (Figure S26).

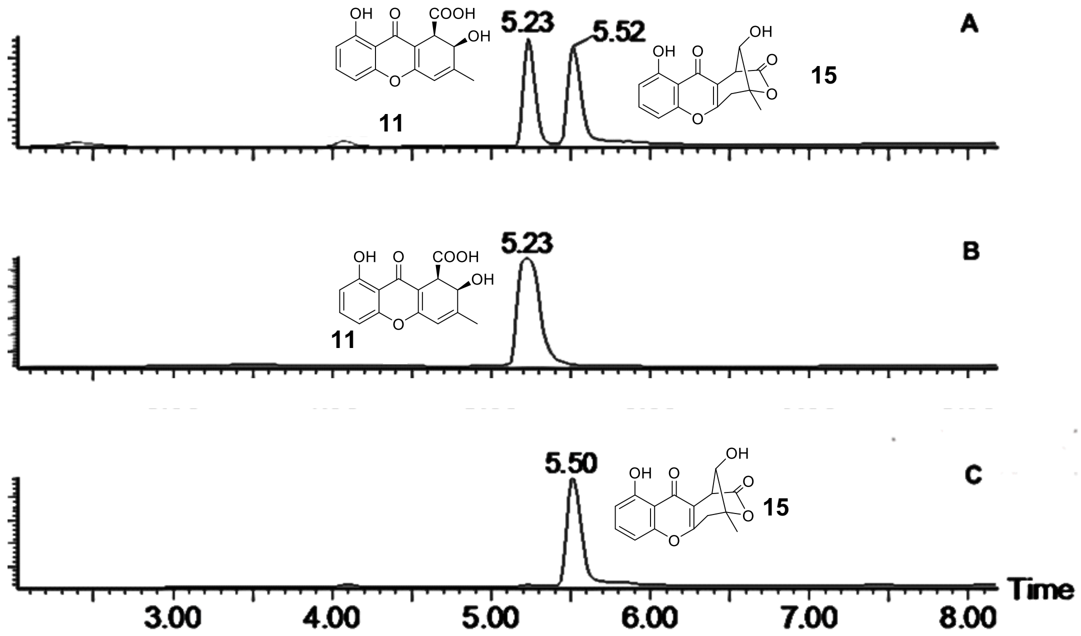

Figure S26. PDA traces of: A – acidified sample of agnestin B (tR 5.5 min) where agnestin A (tR 5.23 min) appeared; B – pure agnestin A sample; C- pure agnestin B sample before acidification (samples were run on a 15 min programme on ZQ).

Agnestin A **11** was also observed to decompose with time. Agnestin B **15** and monodictyxanthone **12** were found in older samples of purified agnestin A (Figure S2.3B).

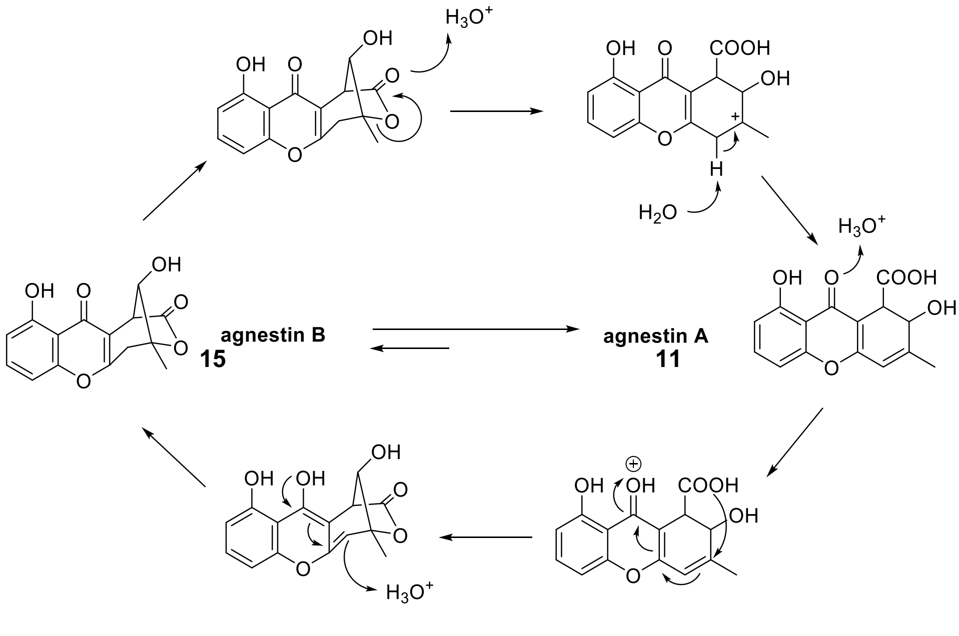

Figure S27. Proposed mechanism of acid-catalyzed rearrangement of agnestin B **15** to agnestin A **11**.

3.0 Sequence Details and Knockout Experiments

3.1 Gene cluster details + Bioinformatics

A database consisting of translated proteins from the *P. variotii* genome was BLAST searched using the MdpG PKS responsible for monodictyphenone biosynthesis in *Aspergillus nidulans*. This analysis identified AgnPKS with 67% identity. The genomic region surrounding *agnPKS* was compared to the *mdp* cluster using the Artemis Comparison Tool (ACT, Figure S28).

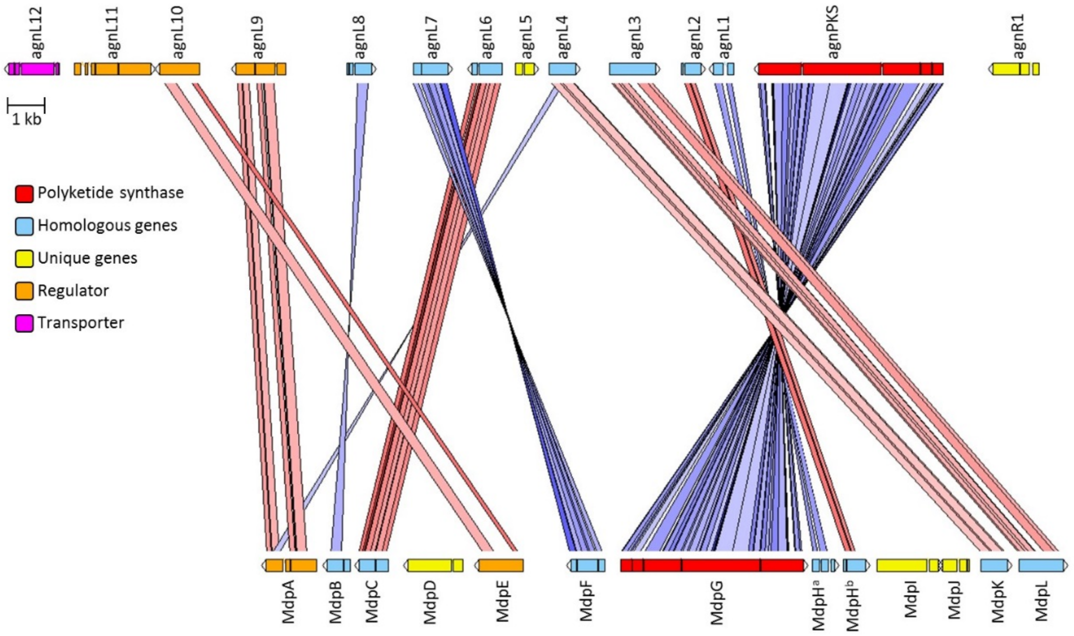

Figure S28. Comparison of the ANmdp gene cluster with the *P. variotii's* PVs6cl30agn cluster using ACT. Red bands indicate regions of similarity on the same strand, blue bands indicate regions of similarity on the opposite strand.

Approximately 50 kb either side of *agnPKS* were analysed for potential ORFs using Softberry FGENESH, along with manual intron/exon analysis. Putative coding sequences were analysed by BLAST against the NCBI ascomycete database, submitted to InterPro to predict protein families, domains and important sites, and also compared to *A. nidulans* monodictyphenone cluster (ANmdp cluster). Artemis Comparison Tool was utilised to compare the *A. nidulans mdp* cluster to the putative agnestin cluster (PVs6c30agn) which allowed identification of homologous genes, and those exclusive to the PVs6c30agn cluster.

The candidate gene cluster was more closely analyzed by pBLAST and the putative proteins encoded by given genes annotated, resulting in a composition of PVs6cl30agn cluster to be proposed as shown on Figure S3.1A and pBLAST annotations listed in Table S6. When the PVs6cl30agn gene cluster was compared with the ANmdp cluster (Table), we were able to differentiate genes that were common

for both clusters (homologous genes) and those that are specific for *P. variotii* cluster (extra genes). The annotated Agn BGC has been uploaded to genBank with accession number MH898872.

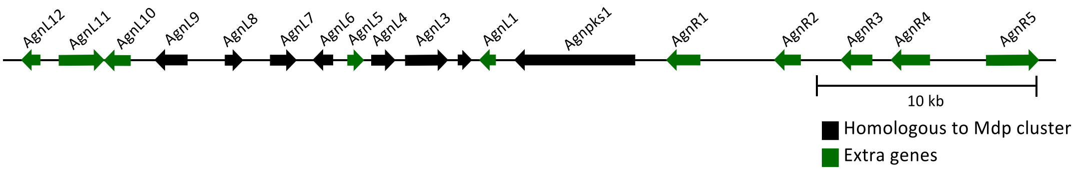

ANmdp cluster (*A. nidulans*)			PVs6cl30 cluster (*P. variotii*)		
Protein	Putative function		Protein	Putative function	Identity [%]
MdpG	Polyketide synthase (nr-PKS)	→	AgnS	nr-PKS	67.38
MdpF	Zn-dependent hydrolase	→	AgnL7	Zn-dependent hydrolase	70.86
MdpC	Ketoreductase	→	AgnL6	Dehydrogenase/Reductase	70.19
MdpB	Dehydratase	→	AgnL8	Dehydratase	55.68
MdpL	Baeyer-villiger oxidase	→	AgnL3	Baeyer-villager oxidase (DUF4243)	42.05
MdpK	Oxidoreductase	→	AgnL4	Oxidoreductase	60.15
MdpH	Putative protein (DUF 1772)	→	AgnL2	Anthrone Oxidase (DUF 1772)	51.88
MdpA	Regulatory gene/O-methyltransferase	→	AgnL9	O-methyltransferase/Regulator	45.62
MdpD	Monooxygenase		AgnL12	MFS transporter	
MdpE	Regulatory gene		AgnL10	C6 transcription factor	
MdpI	Acyl-CoA synthase		AgnL11	C6 transcription factor	
MdpJ	Glutathione S transferase		AgnL5	Putative protein	
			AgnL1	Putative protein	
			AgnR1	NAD-binding	
			AgnR2	BTB/POZ domain containing protein	
			AgnR3	Putative protein	
			AgnR4	RNA binding protein	
			AgnR5	Transcriptional regulatory protein DEP1	

Table S6. PVs6cl30agn gene cluster compared with the ANmdp cluster form *A. nidulans*.

GENE	PUTATIVE FUNCTION	CLOSEST HOMOLOGUE*	IDENTITY	INTERPRO DOMAIN(S)
agnL12	Major facilitator superfamily protein	-		Major facilitator superfamily (PF07690)
agnL11	Transcription factor	-		ZN2/Cyc6 DNA-binding domain (SSF57701), fungal transcription factor (PF04082)
agnL10	Transcription factor	AflR (AAS90003)[164, 165]	30%	ZN2/Cyc6 DNA-binding domain (SSF57701)
agnL9	Regulator	AflJ (BAJ53442) [166]	35%	*O*-methyl transferase domain (PTHR11746), winged helix-turn-helix DNA-binding domain (SSF46785)
agnL8	Dehydratase	SCD1 (BAC79365)[167]	58%	Syctalone dehydratase (PF02982)
agnL7	Hydrolase	AdaB (AEN83888)[168]	50%	Metallo-hydrolase/oxidoreductase (SSF56281)
agnL6	Reductase	Ver1(P50161)[169]	65%	Short-chain dehydrogenase (PR00081)
agnL5		-		NTF2-like (SSF54427)
agnL4		AflX (Q6UEF2)[170]	45%	NAD(P)-binding Rossman-fold domains (SSF51735)
agnL3	Baeyer-Villiger oxidase	AflY (Q6UEF1)[171]	35%	DUF4243 (PF14027)
agnL2	Anthrone oxidase	HypC (B8NI03)[172]	39%	DUF1772 (PF08592)
agnL1		-		EthD domain (PF07110)
AgnPKS	Polyketide synthase	AdaA (AEN83889)[168]	47%	B-ketoacyl synthase domain (SM00825), acyl transferase domain (SM00827), product template domain (TIGR04532), acyl carrier protein domain (PS50075)
agnR1		-		FAD/NAD(P) binding domain (SSF51905)

Table S7. pBLAST annotations of the PVs6cl30agn gene cluster. * closest NCBI experimentally verified homologue

3.2 Transformation and KO procedure

Spores from one plate were inoculated into 100 ml of PDB medium and cultured overnight at 25 ˚C with shaking (200 rpm). The culture was then transferred into 50 ml sterile tubes and spun at 9000 rpm for 10 minutes to separate the mycelia and remove supernatant. Mycelia were then washed with 10 ml of sterile H_2O, spun again at 9000 rpm for 10 minutes and the supernatant removed. The wash step was repeated with 10 ml of 0.7 M KCl. Lysing solution (10 mL) was added to the collected mycelia (0.7 M KCl, 5 $mg \cdot ml^{-1}$ driselase, 5 $mg \cdot ml^{-1}$ Trichoderma lysing enzyme) and incubated at 25 ˚C for 1-2 h with very gentle shaking. The formation of protoplasts was checked regularly under the microscope. The mixture was then filtered through a sterile miracloth and spun at 3000 x g for 3 minutes to remove supernatant. Collected protoplasts were washed with 10 ml 0.7 M KCl, 50 mM $CaCl_2$ and supernatant removed. Then, approx. 600 µl of 0.7 M KCl, 50 mM $CaCl_2$ were added. 200µl aliquots taken out into fresh sterile 10 ml tubes, 10 µl of DNA fragments (see below) and 50 µl PEG solution (25% *w/v* PEG 3350, 50 mM $CaCl_2$, 10 mM Tris-HCl pH 7.5) added and the mixture incubated on ice for 20 min. Next, 500 µl of PEG solution was added into each tube and further incubated at 25 ˚C for 5 mins. 200 µl were then plated out on PDA plates containing 1 M sorbitol and incubated overnight at 25 ˚C. On the next day, a 10 ml overlay of PDA containing enough hygromycin B to make the overall concentration of the plate 150 $\mu g \cdot ml^{-1}$ was added. Colonies that grew through were picked onto secondary, hygromycin-containing PDA plates and once sporulated, were streaked to single colonies transferred onto tertiary plates.

P. variotii gDNA was used as a template for amplification of the target gen*e* - *LHS* (PCR1) and – RHS (PCR2) fragments. The LHS (PCR2) and RHS (PCR3) fragments of the HygR cassette were amplified from pTHygGS-eGFP plasmid. The knockout Left-Fragments (PCR5) and Right-Fragmentss (PCR6) were prepared by fusion PCR[7] (KOD Hot Start Polymerase). The genetic testing for LHS (Test1) and RHS (Test2) integration, as well as presence of intact gene (test3), was carried out on gDNA extracted from transformants.

**Fragment**	**Forward Primer**	**Reverse Primer**	**Size [bp]**
**PVs6cl30agnL4**			
**PCR1**	CCCTGACAACTCCCTACTAT	CACTAGAGGATCCCCATCATG<br>GCTGCCTTCGTAGATCTGTA	**539**
**PCR2**	TCCGAGGGCAAAGGAATAGGTCGACCATCTTCATCAAGC	CCAATACAATGAGAGTCAGG	**573**
**PCR5**	CCCTGACAACTCCCTACTAT	CGTCAGGACATTGTTGGAG	**3457**
**PCR6**	GCTTTCAGCTTCGATGTAGG	CCAATACAATGAGAGTCAGG	**1475**
**PVs6cl30agnL3**			
**PCR1**	CTGGTTGCACCGCTATCTTC	ACTAGAGGATCCCCATCATGCATCGCGCATCTTGTTGCCA	**1835**
**PCR2**	CACATCTCCACTCGACCTGTTTCTTCACCGTGCTGATCC	TGGTGGCCATAGTGGTACAT	**1527**
**PCR5**	CTGGTTGCACCGCTATCTTC	CGTCAGGACATTGTTGGAG	**4611**
**PCR6**	CTGTCGAGAAGTTTCTGATCG	TGGTGGCCATAGTGGTACAT	**3297**
**PVs6cl30agnR1**			
**PCR1**	TGCTTTCTCACATACTGGAG	ACTAGAGGATCCCCATCATGCATTCATACAGGGTCACCTC	**944**
**PCR2**	TCCGAGGGCAAAGGAATAGCTCGAGTTTCGTCATGGTAG	GTGCGTCTGGCTATTTCCTT	**622**
**PCR5**	TGCTTTCTCACATACTGGAG	CGTCAGGACATTGTTGGAG	**3719**
**PCR6**	GCTTTCAGCTTCGATGTAGG	GTGCGTCTGGCTATTTCCTT	***1523***
**PVs6cl30agnPKS**			
**PCR1**	ATACTCCTCCAACCAACTGC	CACTAGAGGATCCCCATCATGG<br>GCGACGACAATTATACGAC	**1765**
**PCR2**	CACATCTCCACTCGACCTGGC<br>ATCGTTAGCAATGATCCG	CTTTCGGGAACTTCACAACC	**1836**
**PCR5**	ATACTCCTCCAACCAACTGC	CGTCAGGACATTGTTGGAG	**4540**
**PCR6**	CTGTCGAGAAGTTTCTGATCG	CTTTCGGGAACTTCACAACC	**3606**
**Hygromycin Resistance Cassette**			
With TtrpC (agnPKS, agnL3)			
**PCR2**	CATGATGGGGATCCTCTAGTG	CGTCAGGACATTGTTGGAG	**2796**
**PCR3**	CTGTCGAGAAGTTTCTGATCG	CAGGTCGAGTGGAGATGTG	**1789**
**Without TtrpC (agnL4, agnL5, agnR1)**			
**PCR3**	GCTTTCAGCTTCGATGTAGG	CTATTCCTTTGCCCTCGGA	**921**

Table S8. Primer sequences used for constructs preparation for knocking out genes from PVs6c30agn gene cluster.

3.3 ΔAgnPKS

Disruption of the *agnPKS* gene led to non-production of agnestins, monodictyphenone and related metabolites by *agnΔPKS* mutant (Figure S29), confirming that all those compounds shared polyketide precursor and that we have identified the correct gene cluster.

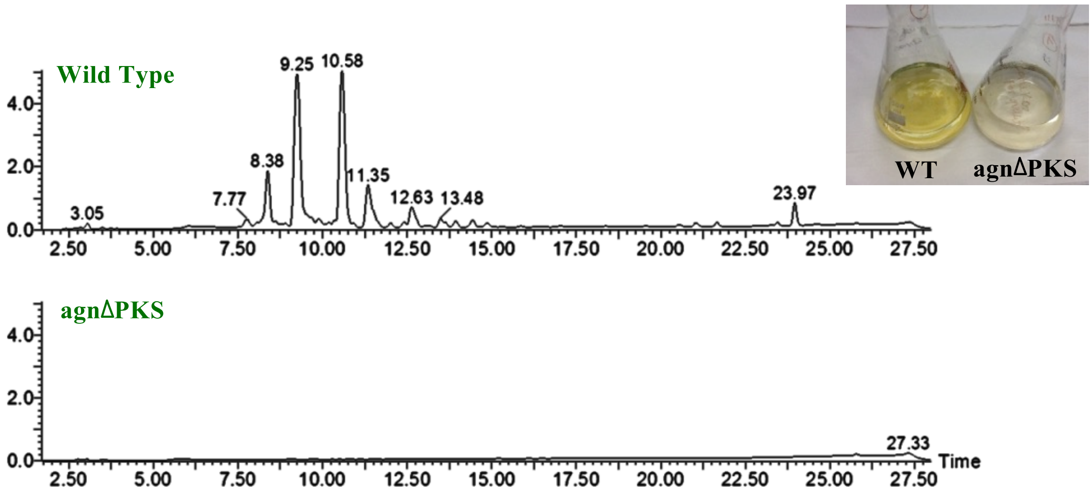

Figure S29. PDA chromatograms of WT P. variotii (top) and agnΔPKS mutant (bottom) extracts.

3.4 ΔAgnL4

The *agnL4* gene was targeted for disruption. When cultured for metabolite extraction and analysed by the LCMS, the PCR-confirmed mutant was found to accumulate emodin but not chrysophanol (Figure S30).

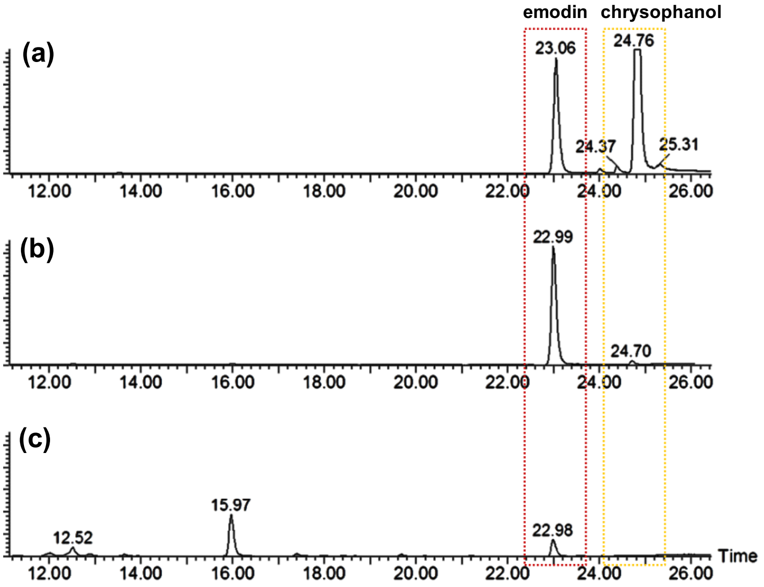

Figure S30. ELSD traces of extracts from the culture liquid (**c**) and (**b**) mycelia extract from the agnΔH mutant, compared with mycelia extract from the agnΔL3 mutant (**a**).

3.5 ΔAgnL3

One ΔAgnL3 transformant was confirmed by PCR analysis to carry the correct mutation and was selected for further testing. LCMS analysis of the secondary metabolites extracted from the mutant showed that neither agnestins nor monodictyphenone were produced.

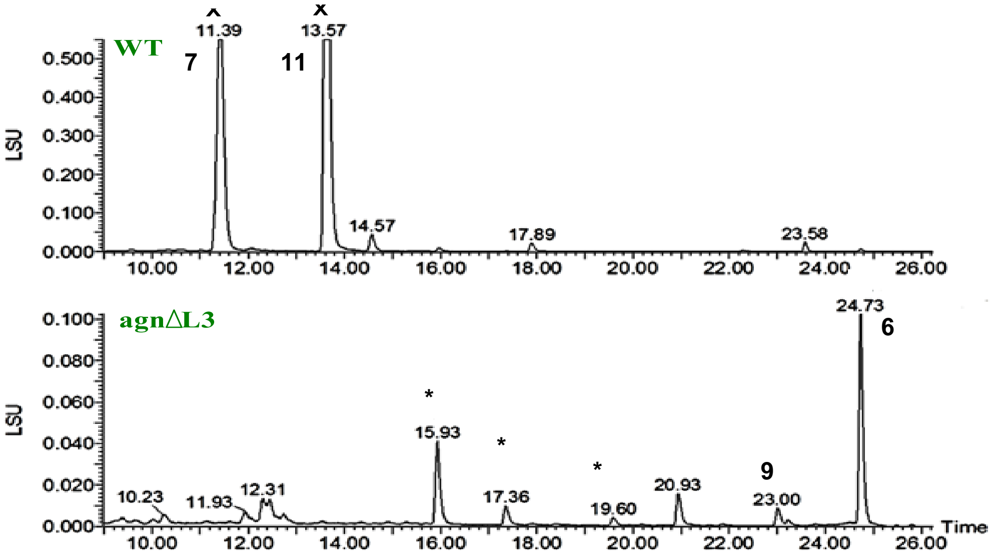

Figure S31. ELSD chromatogram of culture liquid extract from WT *P. variotii* (top) and from the agnΔL3 mutant (bottom). * Unrelated maleidrides.

A sample of mycelia extract (16 mg out of 1.5 g of crude extract dissolved in 6 ml of MeCN) was subjected to HPLC-purification, which yielded 6.5 mg of emodin **9** and 7.6 mg of chrysophanol **6**, corresponding to the production of 677 mg and 791 mg respectively per litre culture.

Two compounds (t_R 22.9 and 24.7 min) were produced in large (when compared with typical yields of other metabolites) amounts by the *agnΔL3* mutant. For the metabolite eluting at t_R 22.9 min, the ESIMS spectra confirming molecular mass could be obtained in the chromatographic conditions (peak of *m/z* 269.5 in $^{-}$ESIMS and 271.5 in $^{+}$ESIMS), however metabolite eluting at t_R 24.7 min did not ionize well enough to give the ESIMS signal. Both compounds had very interesting UV spectra,

confirming their xanthone affiliation. Preparative TLC yielded two colourful compounds (Figure S32).

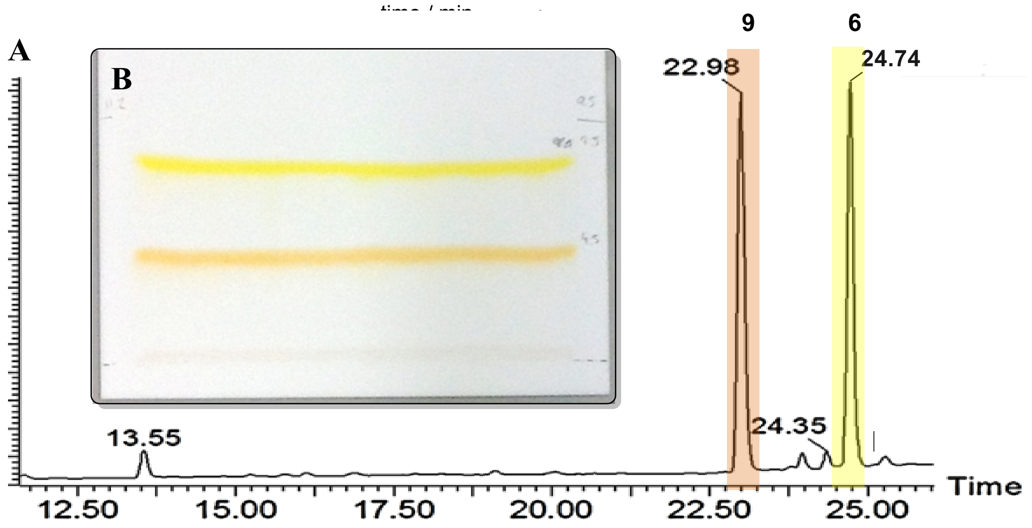

Figure S32. A - PDA traces of mycelial extract from the *agnΔL3* mutant, showing almost entirely peaks of chrysophanol **6** and emodin **9** ; B – a preparative TLC plate.

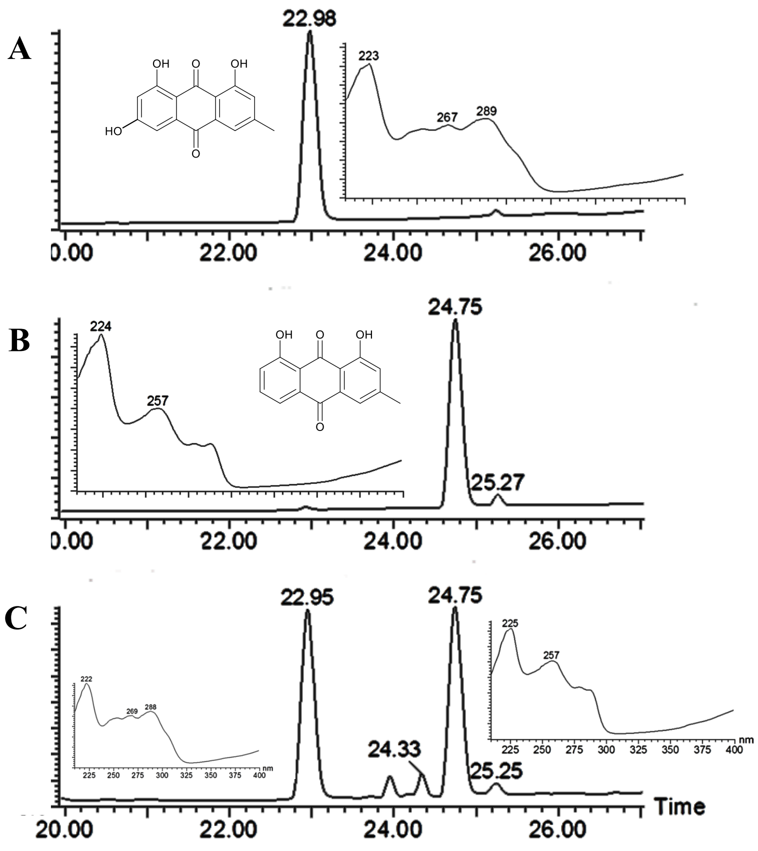

Figure S33. Comparison of PDA chromatograms and UV spectra of: **A** - standard emodin; **B** - standard chrysophanol; C - agnΔL3 mutant mycelia extract.

3.6 ΔAgnR1

More than 50 ΔAgnR1 transformants were isolated after the use of a standard bipartite KO procedure (Figure S34). The *agnR1* gene knockout transformants were analysed by various PCR reactions using genomic DNAs from the transformants and *P. variotii* WT.

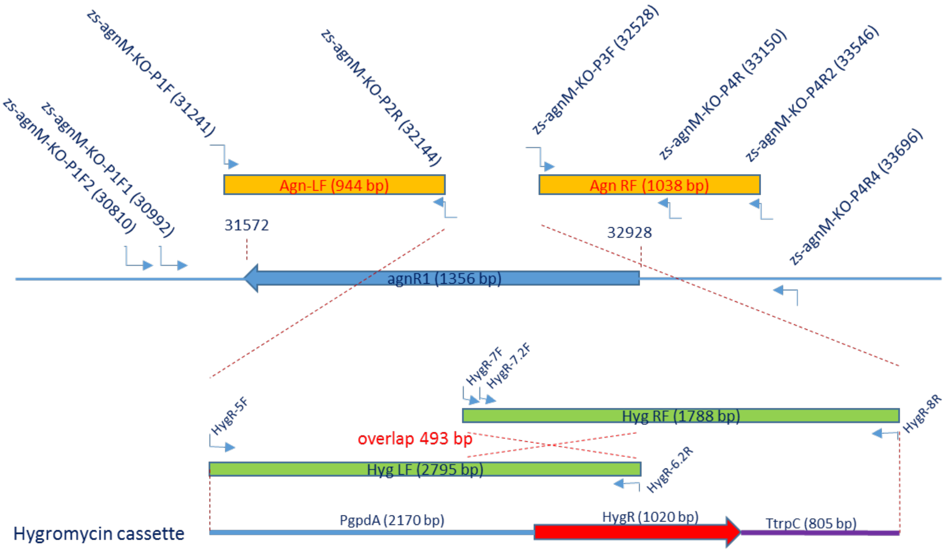

Fig S34. agnR1 gene knockout strategy and primer designs.

Table S7. Primer sequences used for agnR1 knockout

Primer name	Sequence (5′-3′)	Length (nt)	Tm (°C)
zs-agnM-KO-P1F	gccaactttgtacaaaaaagcaggctccTGCTTTCTCACATACTGGAG (The lower case is attR1 homologous sequence)	20	58.0
zs-agnM-KO-P2R	cgaaagatccactagaggatccccatcatgCATTCATACAGGGTCACCTC (The lower case is complimentary sequence of HygR 5F)	20	59.4
zs-agnM-KO-P3F	gcgcccactccacatctccactcgacctgCTCGAGTTTCGTCATGGTAG (The lower case is complimentary sequence of HygR 8R	20	59.9
zs-agnM-KO-P4R	tttgtacaagaaagctgggtcggcgcgcccGTGCGTCTGGCTATTTCCTT (The lower case is attR2 homologous sequence)	20	63.1
zs-agnM-KO-P4R2	tttgtacaagaaagctgggtcggcgcgcccATCATTGCTCATCTGACGAC (The lower case is attR2 homologous sequence)	20	60.3
zs-agnM-KO-P4R3	AGACCGATGATGTTCGTGTC	20	62.3
zs-agnM-KO-P4R4	CGATAGGGATAAGAAGCTCGTG	22	63.7
zs-agnM-KO-P1F2	TCTCTCCGATATTAACAGCGG	21	62.9
Hyg-5F	CATGATGGGGATCCTCTAGTG	21	63.3
Hyg-6R	CTCCAACAATGTCCTGACG	19	61.8
Hyg-6.2R	CGTCAGGACATTGTTGGAG	19	61.8
Hyg-7F	CTGTCGAGAAGTTTCTGATCG	21	61.6
Hyg-7.2F	TTCGACAGCGTCTCCGACCT	20	69.6
Hyg-8R	CAGGTCGAGTGGAGATGTG	19	62.1

The first PCR is to confirm presence and correct recombination of the two fragments of hygromycin cassette using primer hyg 7.2F and hyg 6.2R (Fig S35). Results show Pv-2, Pv-3, Pv-26, Pv-44 and Pv-47 are negative. All the other transformants tested positive.

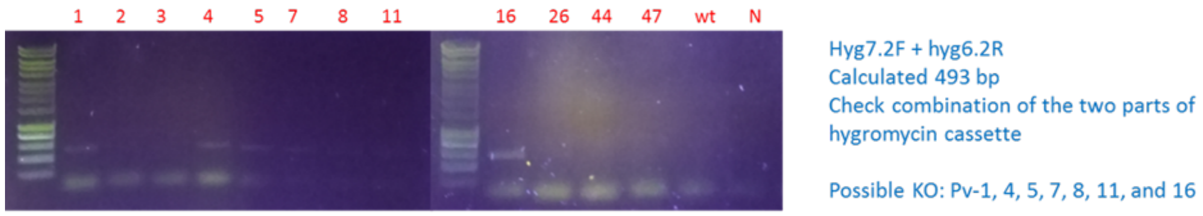

Fig S35. PCR results for hygromycin cassette using primers hyg7.2F and hyg 6.2R.

PCR for intact *agnR1* was carried out using agnM-KO-P1F and agnM-KO-P4R. PCR reaction for agnM1F+agnM4R was set to amplify only intact *agnR1* while the *agnR1* gene with hygromycin inserted would be too large to be amplified (6 kb). The results show Pv-8 and Pv-11 have the expected insertion (Figure S36).

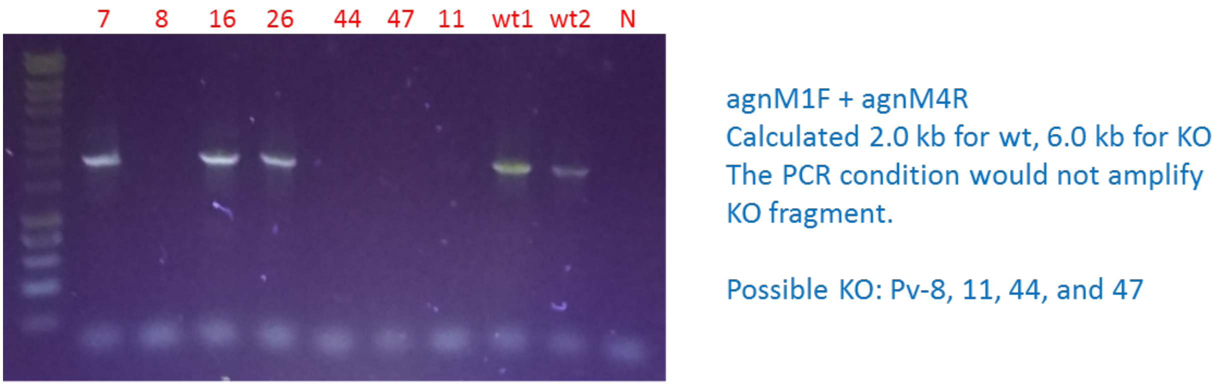

Fig S36. Detection of intact agnR1 gene by PCR. Primer pair used are agnM-KO-P1F and agnM-KO-P4R.

The next PCR reaction (Figure S37) is to confirm the presence of right-hand KO fragment in the genome. Results show Pv-7, Pv-8, Pv-11, Pv-16 and Pv-26 all contain the right-hand KO fragment. From these results Pv-44 and Pv-47 can be confirmed as contamination. Pv-7, Pv-16 and Pv-26 are mistargeting transformants. Pv-8 and Pv-11 are likely agnR1 KO transformants.

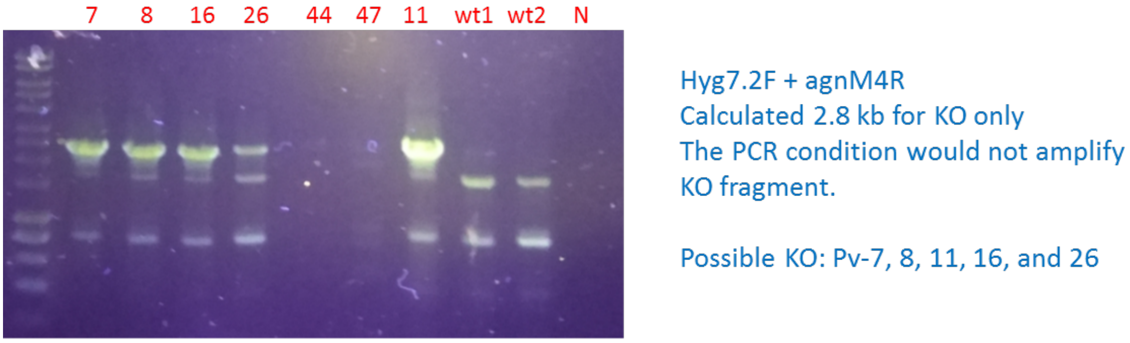

Fig S37. Detection of intact *agnR1* by PCR. Primer pair used are hyg7.2F and agnM-KO-P4R.

To further confirm the correct targeting position of the KO fragments new PCR primers were designed at both 5’- and 3’- end position outside of the KO sequence. These PCR reactions would confirm the correct targeting position in *agnR1* by the two KO fragments. PCR for the right-hand KO fragment shows Pv-8 and Pv-11 are correct *agnR1* recombinants while all the other transformants have a smaller PCR product (Fig S38). PCR for left hand KO fragment in correct position were carried out using agnM-KO-1F2 and hyg 6.2R. Only Pv-8 and Pv-11 show correct result clearly (Fig S39).

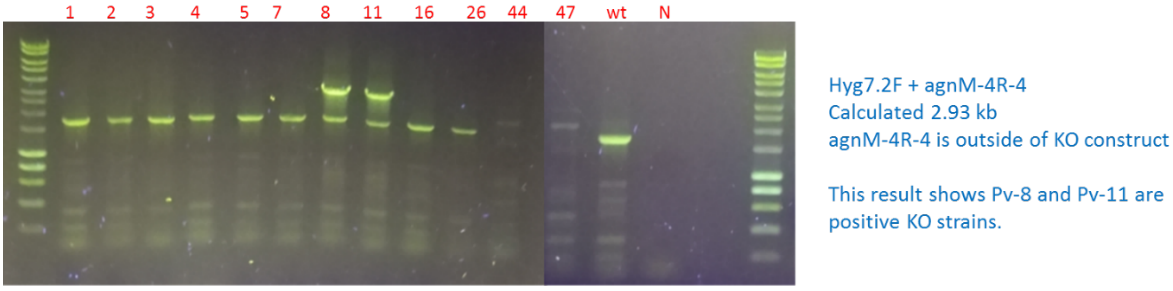

Fig S38. PCR reaction for agnR1 right-hand KO fragment.

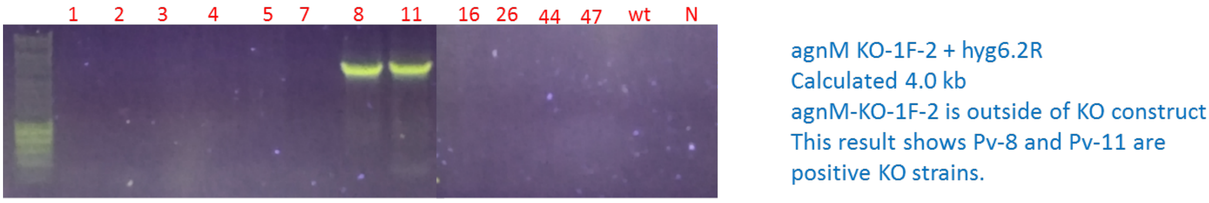

Fig S39. PCR reaction for agnR1 right-hand KO fragment.

Finally strain Pv-11, Pv-8 and Pv7 were grown under producing conditions, extracted and examined by LCMS (Figure S40 KO strains Pv-11 and Pv-8 still produce WT compounds, while Pv-7 with incorrect integration produces nothing, possibly indicating disruption of *agnPKS*.

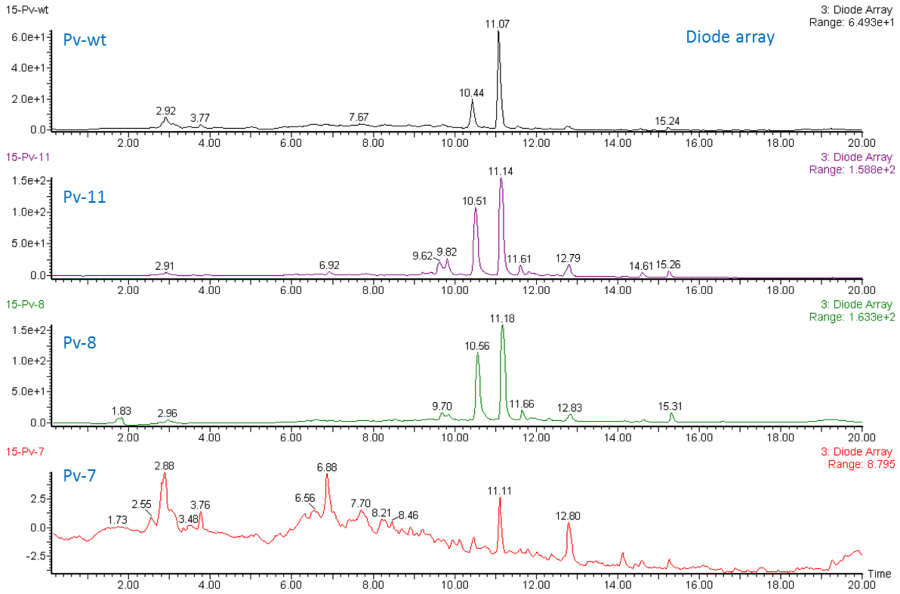

Fig S40. Diode array traces from agnR1 KO strains Pv-8, Pv-11 and P. variotii WT.

4.0 References

1. J. Sanchez, R. Entwistle, J.-H. Hung, J. Yaegashi, S. Jain, Y.-M. Chiang, C. Wang, and B. Oakley, *J. Am. Chem. Soc.*, 2011, **133**, 4010–4017.
2. R. Gonçalves, E. Silva, N. Hioka, C. Nakamura, M. Bruschi, and W. Caetano, *Nat. Prod. Res.*, 2018, **32**, 366–369.
3. T. Asai, S. Otsuki, H. Sakurai, K. Yamashita, T. Ozeki, and Y. Oshima, *Org. Lett.*, 2013, **15**, 2058–2061.
4. A. Krick, S. Kehraus, C. Gerhäuser, K. Klimo, M. Nieger, A. Maier, H.-H. Fiebig, I. Atodiresei, G. Raabe, J. Fleischhauer, and G. König, *J. Nat. Prod.*, 2007, **70**, 353–360
5. D. Pockrandt, L. Ludwig, A. Fan, G. König, and S. Li, *ChemBioChem*, 2012, **13**, 2764–2771.
6. C. Li, J. Zhang, C. Shao, W. Ding, Z. She, and Y. Lin, *Chem. Nat. Compd.*, 2011, **47**, 382–384.
7. N. A. Shevchuk, A. V. Bryksin, Y. A. Nusinovich, F. C. Cabello, M. Sutherland and S. Ladisch, *Nuc. Acids Res.*, 2004, **32**, e19.
